# Melatonin Uncouples Ripening Promotion from Softening and Enhances the Polyphenol Profile in Postharvest ‘Reineta’ Apples (*Malus domestica*)

**DOI:** 10.3390/plants15193038

**Published:** 2026-10-04

**Authors:** Antía Verde, Jesús M. Míguez, Mercedes Gallardo

**Affiliations:** 1 Departamento de Biología Vegetal y Ciencias del Suelo, Universidade de Vigo, 36310 Vigo, Spain; averde@uvigo.es; 2 Departamento de Biología Funcional y Ciencias de la Salud, Universidade de Vigo, 36310 Vigo, Spain; jmmiguez@uvigo.es

**Keywords:** *Malus domestica*, melatonin, ethylene, postharvest ripening, firmness, phenolic compounds

## Abstract

Melatonin regulates fruit ripening and postharvest quality, but its effect on the phenolic profile of apples and its interaction with ethylene remain unclear. We evaluated exogenous melatonin (10, 50, 500 µM), 1-aminocyclopropane-1-carboxylic acid (ACC) and their combination on quality parameters and the polyphenolic profile of ‘Reineta’ apples (*Malus domestica*) during 35-day postharvest ripening at 22 ± 2 °C. Sugars, firmness, color and ethylene production were determined, together with total phenols, flavonoids and eleven polyphenols quantified by HPLC-DAD in skin and flesh. Melatonin accelerated ripening—advancing ethylene production, increasing sugars and promoting color development—while significantly delaying softening. ACC reproduced the ethylene and color responses but did not modify firmness or phenolic content, whereas the melatonin–ACC combination synergistically enhanced ethylene production without compromising texture or coloration. Melatonin attenuated the postharvest decline in phenols, flavonoids and individual polyphenols, mainly in skin and on chlorogenic acid, procyanidin B2 and quercetin glycosides; 50 and 500 µM were most effective. Melatonin thus uncoupled rising ethylene from firmness loss within the climacteric cascade, improving fruit quality through a protective mechanism additional to the ethylene pathway, plausibly linked to its antioxidant capacity, and making this indoleamine a promising tool for modulating apple ripening after harvest.

## 1. Introduction

Apple (*Malus domestica* Borkh.) is one of the most widely consumed edible fruits worldwide and is broadly available commercially. It possesses high nutritional value due to its content of sugars, vitamins, and minerals and also constitutes an important source of antioxidants, including flavonoids, anthocyanins, and other phenolic compounds [1,2,3]. Polyphenols are plant secondary metabolites with strong antioxidant capacity, able to scavenge reactive oxygen species (ROS) and other free radicals, thereby preventing oxidative damage [4] and contributing to the enhancement of antioxidant capacity in plant tissues [5].

The content and composition of polyphenols in apples are of considerable interest because of their contribution to both fruit sensory quality and antioxidant capacity, which has led to their association with beneficial health properties [6,7,8,9]. Indeed, comparative studies rank apples among the fruits with the highest antioxidant potential [10,11]. In addition, the peel has been reported to contain higher levels of phenolic compounds than the flesh, including some metabolites exclusive to this tissue [1,12]. In general terms, the main groups of polyphenols present in apples include: (i) hydroxycinnamic acids, mainly chlorogenic acid; (ii) flavanols, such as (+)-catechin and (−)-epicatechin derivatives, as well as procyanidins; (iii) dihydrochalcone derivatives; (iv) flavonols, mainly quercetin and kaempferol derivatives; and (v) anthocyanins [13,14,15]. Several studies have demonstrated that the profile of phenolic compounds may vary depending on fruit developmental stage, cultivar, climatic conditions, irradiance, water availability, and geographical location [12,15,16]. However, fewer studies have focused on variations in these compounds during fruit growth and ripening. In this regard, high concentrations have been reported at early developmental stages followed by a progressive decrease during fruit development [17,18], as well as maximum levels prior to ripening, followed by a subsequent decline [15].

Fruit ripening involves a series of physiological, biochemical, and organoleptic changes associated with the formation of the mature fruit [19,20,21]. During this process, changes occur in coloration, tissue softening, accumulation of sugars and organic acids, and synthesis of aromatic compounds responsible for characteristic flavor and aroma [22,23,24]. Apple is a climacteric fruit characterized by an increase in respiration associated with enhanced ethylene production at the onset of ripening, this phytohormone being the main regulatory signal of the process [25]. Nevertheless, other hormones are also involved in ripening control [26], including abscisic acid (ABA) [27], auxin [28], and melatonin [21].

Melatonin is an indoleamine present in plants [29], whose concentrations vary widely among species and organs depending on physiological and environmental conditions [30,31]. Among its main functions, melatonin stands out for its strong antioxidant capacity, neutralizing free radicals and ROS [32,33], which has led several authors to propose a relevant role during fruit development and ripening, processes marked by intense metabolic activity and enhanced ROS generation. In line with findings reported in other fruits such as cucumber, grape, tomato, and cherry [34,35,36,37], melatonin has been suggested to act as an antioxidant and ROS-scavenging molecule during apple growth and ripening [38]. Beyond this, melatonin regulates diverse processes of plant growth and development, including senescence and fruit ripening [21,39,40,41,42,43], and modulates the biosynthetic pathways of other hormones, notably ethylene [21,44,45,46,47].

The role of melatonin in fruit ripening and senescence is well supported [48,49], although its effects are species-dependent: it promotes ripening in tomato by increasing ethylene production [21], whereas in banana and pear it delays senescence by reducing ethylene synthesis [50,51], with ripening-delaying effects also reported in other climacteric fruits [52,53,54,55,56] and variable responses in non-climacteric ones [5,48,57]. In apples, preharvest melatonin application to branches bearing green fruits stimulated ripening—advancing ethylene production and accelerating coloration and sugar accumulation—while delaying senescence and mitigating softening [58]; subsequently, postharvest studies in ‘Golden’ apples showed that melatonin advanced ethylene production through induction of MdACS1 and MdACO1, correlating with enhanced coloration, sweetness and firmness retention [59]. Melatonin may also interfere with secondary metabolism, increasing phenolic content and antioxidant capacity and thereby improving fruit quality [60], as described in sweet cherry [61,62], blackberry [63], strawberry [64] and pomegranate [65]; in tomato it stimulates anthocyanin-biosynthesis genes [45], and in grape it increases phenols, flavonoids and anthocyanins, possibly through interactions with the ethylene pathway [5]. Ethylene itself promotes the biosynthesis of phenolic compounds and flavonoids, which contribute to both antioxidant properties and sensory characteristics [66].

In apples, as in other climacteric fruits, the rise in ethylene production that drives ripening is normally accompanied by progressive softening, so that ripening and firmness loss advance together. In this context, we use the term uncoupling to denote the capacity of melatonin to allow ethylene to rise and promote ripening-related changes—sugar accumulation and color intensification—without the concomitant loss of firmness that usually accompanies them.

Despite these advances, and although the effect of melatonin on the ripening of various fruits has been described, no study has yet characterized its effect on the content and profile of phenolic compounds during apple ripening, nor its possible relationship with ethylene. To address this gap, the aim of the present study was to evaluate the effect of melatonin application on the profile of phenolic compounds—distinguishing skin from flesh—during different stages of postharvest ripening in ‘Reineta’ apples, and to analyze its possible relationship with ethylene synthesis, together with the implications of these effects for fruit quality.

## 2. Results

### 2.1. Evolution of Fruit Quality Parameters During Postharvest Ripening: Effect of Melatonin and ACC Treatment

The evolution of the fruit quality parameters (color variation, fruit firmness and sugar content), as well as ethylene production, was monitored in ‘Reineta’ apples over 35 days of postharvest ripening. The results are shown in Table 1.

In untreated fruit, total sugar content increased significantly during postharvest ripening (days 0 to 35). ACC treatment produced no significant changes relative to the control at any sampling time. Melatonin treatments alone significantly increased sugar levels at day 35 (MEL 50 µM), an effect also observed when MEL 50 µM was applied in combination with ACC at the same time. At MEL 500 µM, sugar values were numerically higher than in the control, although the differences were not statistically significant.

In the control group, fruit firmness decreased during the ripening of ‘Reineta’ apple. Melatonin treatments alone significantly delayed softening, especially at day 35. ACC did not significantly affect firmness, but its combination with each of the melatonin concentrations produced fruit with a higher degree of firmness at day 35, reaching values similar to those obtained with melatonin alone.

The apple color changed slightly until day 14 and abruptly thereafter until day 35, shifting from the initial green (day 0) to a warm background color with the reddish striping characteristic of the ‘Reineta’ cultivar (day 35). All treatments with MEL (10, 50 and 500 µM), ACC and MEL + ACC induced a significant increase in color variation relative to the untreated group (control) at each sampling time (7, 14 and 35 days). Moreover, the presence of MEL, alone or combined with ACC, produced a greater color variation at day 35 compared with ACC applied alone.

As for ethylene production, previous work described typical climacteric behavior in ‘Reineta’ apples [59]. Although the temporal resolution of this study (0, 7, 14 and 35 days) did not allow the climacteric maximum to be captured in all groups, our previous work on the same cultivar [59] showed that the peak occurs at around day 21; the data presented here therefore correspond to the ascending (day 14) and post-climacteric or incipient-senescence (day 35) phases, which are sufficient to demonstrate the effects of the treatments on these key quality parameters. In the control, ethylene was undetectable at days 0 and 7, increased at the onset of ripening (day 14) and reached levels more than 10-fold higher at day 35 relative to those of the previous sampling at day 14. With the MEL, ACC and MEL + ACC treatments, ethylene production was already observed at day 7, with levels increasing significantly at days 14 and 35 relative to the control group. The MEL + ACC combination showed an even greater effect at day 14, increasing ethylene production 2- to 3-fold compared with the treatments applied separately.

### 2.2. Evolution of Total Phenolic and Flavonoid Contents During Postharvest Ripening: Effect of Melatonin and ACC Treatment

Total phenolic and flavonoid contents were analyzed in skin and flesh. The levels of both components were higher in the skin: two- to three-fold for phenolics (Figure 1) and approximately three-fold for flavonoids, compared with the flesh (Figure 2). In the control group, total phenolic content decreased significantly at day 7 in both tissues relative to the green fruit (day 0), increasing subsequently during ripening (days 14 and 35), especially in the flesh, where the increase was significant at day 35, although without recovering the initial levels.

Three-way ANOVA showed a significant effect of melatonin on total phenolic content in both skin and flesh (*p* < 0.001) over the course of ripening (Table 2). ACC treatment also produced a significant effect, although only in the flesh (*p* < 0.001), being affected by time. In this tissue, a significant interaction between the two treatments, ACC and MEL, was observed (*p* = 0.021).

Melatonin treatment alone kept phenolic content high throughout the sampling period, with values similar to those present in the green fruit. At day 7, all melatonin doses increased phenolic content, whereas at day 14 this effect was limited to the 50 and 500 µM doses (Figure 1A), compared with the respective control group. In the flesh, a significant increase was observed with the higher doses at days 7 and 14 (Figure 1B). At day 35, in both skin and flesh, phenolic levels under MEL were numerically higher than in the control, without reaching statistical significance. ACC treatment showed a profile similar to the control for total phenolics, whereas the combination of ACC and MEL produced high phenolic levels at day 7, similar to those obtained with MEL alone. At the remaining times analyzed, phenolic levels under the combined treatment were numerically higher than in the control, without reaching significance.

In the absence of treatment, total flavonoid content also decreased significantly in the skin at the onset of ripening (day 7) relative to the time of harvest, undergoing a subsequent recovery such that at day 35 the levels were similar to those of the green fruit (Figure 2A). Total flavonoid content in the flesh showed a behavior opposite to that observed in the skin (Figure 2B). Thus, levels increased significantly at day 7 (1.47-fold relative to day 0) and declined gradually during ripening.

Three-way ANOVA revealed a significant effect of both melatonin and ACC on total flavonoid content, in both skin and flesh (*p* < 0.001); whereas the effect of melatonin remained independent of ripening time, in the flesh, ACC showed a significant interaction with time (*p* < 0.001).

The analysis within each ripening time showed that MEL at the 50 and 500 µM concentrations significantly increased flavonoid content in the skin at day 7 (*p* < 0.001 vs. control) (Figure 2A). In the flesh, MEL increased flavonoids significantly at day 35 with the higher doses, while at the other sampling times the values did not differ significantly from the control (Figure 2B). ACC treatment produced a stimulatory effect on flavonoid concentration in the skin, being significant at days 7 and 14 relative to the untreated control (*p* < 0.001). In the flesh, this effect was not observed, with values similar to those of the control group. The combination of MEL and ACC resulted in even higher flavonoid levels in the skin relative to the control group; this effect was also significant at day 7 relative to the ACC-treated group. In the flesh, the combined treatment increased flavonoid levels at day 14 (*p* < 0.05 vs. control), but not relative to the ACC group. At day 35, melatonin did not alter flavonoid levels when combined with ACC, in contrast to the stimulatory effect observed when applied alone.

### 2.3. Evolution of Total Polyphenol Content During Postharvest Ripening: Effect of Melatonin and ACC Treatments

The technique used allowed the quantification of eleven of the main individual phenolic compounds present in both the skin and the flesh of the apple. Based on their chemical structure, these compounds were grouped into four categories: (i) simple phenolic acids (chlorogenic, caffeic, *p*-coumaric and *o*-coumaric acids); (ii) flavanols (catechin, procyanidin B2 and epicatechin); (iii) flavonols (quercetin, rutin and avicularin); and (iv) dihydrochalcone derivatives (phloridzin). In addition, a total polyphenol index was established for each category, calculated as the sum of the individual compounds in each sample and sampling time, in both skin (Table 3) and flesh (Table 4). This index made it possible to assess the relative contribution of each polyphenolic category to the changes observed during postharvest ripening, as well as the temporal evolution of the total and partial content of the quantified polyphenols.

In the absence of treatment and in the green fruit (day 0), flavanols constituted the main phenolic group in the skin, representing 74.33% of the total quantified polyphenols. Phenolic acids contributed 15.41%, whereas dihydrochalcones and flavonols contributed to a lesser extent, with 6.18% and 4.08%, respectively. In the flesh, although flavanols were also the predominant compounds (53.60%), the contribution of phenolic acids was considerably higher than in the skin, reaching 42.16% of the total. By contrast, dihydrochalcones (3.56%) and flavonols (0.67%) showed a reduced contribution. During postharvest ripening, the skin maintained the same distribution pattern observed at day 0. However, in the flesh of the ripe apple (day 35), phenolic acids became the major group, representing 56.68% of the total, followed by flavanols (40.84%). The contribution of dihydrochalcones and flavonols was much lower: 2.1% and 0.35%, respectively.

In untreated apples (control), total phenolic-compound content at harvest (day 0) was approximately three-fold higher in the skin (2483.72 µg/g) than in the flesh (844.37 µg/g). At the onset of ripening (day 7), polyphenol content decreased significantly in both tissues, with levels 2.19-fold lower in the skin and 2.48-fold lower in the flesh relative to the green fruit. During days 14 and 35, phenolic content increased in both tissues relative to day 7, although without recovering the initial levels. Compared with day 0, the values quantified in the skin at day 35 were 1.45-fold lower (1684.81 µg/g), whereas in the flesh the decrease was 2.08-fold (413.19 µg/g).

The treatments tested in this study—MEL (10, 50 and 500 µM), ACC (1 mM) and the MEL + ACC combination—did not significantly modify the relative contribution of the different polyphenol categories to the total content. Nevertheless, they did produce significant effects on total polyphenol content in both skin and flesh (Table 3 and Table 4). As in the control group, all treatments caused a decrease in total polyphenol content and in each of its components at the onset of ripening (day 7) relative to the green fruit. This reduction was more pronounced in fruit treated with ACC (skin: 1.74-fold; flesh: 1.98-fold) and with the lowest melatonin dose (MEL 10 µM; skin: 1.83-fold; flesh: 2.14-fold). By contrast, the higher melatonin doses (50 and 500 µM) alone, as well as their combination with ACC, attenuated this decrease and reached levels significantly higher than the control in both tissues at day 7.

As ripening progressed (days 14 and 35), all treatments promoted significant increases in total polyphenol content in the skin and, in certain cases, in the flesh. ACC treatment significantly increased phenolic content in the skin at day 14; this effect was maintained at day 35, with levels higher than the control at both times. In the flesh, however, this increase was only evident at day 35. As for melatonin applied alone, all concentrations produced a positive effect on total polyphenol content in the skin during days 14 and 35. This effect was more marked with the 50 and 500 µM doses and with the MEL + ACC combination, reaching levels similar to those of the green fruit as early as day 14, which remained high until the end of ripening.

In the flesh, all melatonin doses increased total polyphenol content from day 14 onward, this effect being maintained until the ripe fruit, although in no case reaching the initial levels of day 0. The combination of MEL 50 µM and ACC produced stimulatory effects at day 35, reaching levels significantly higher than those obtained with ACC applied alone. However, unlike what was observed in the skin, none of the melatonin or MEL + ACC treatments restored in the flesh the polyphenol levels present in the green fruit.

### 2.4. Polyphenolic Profile During Postharvest Ripening: Effect of Melatonin and ACC Treatments

Individual analysis of the phenolic compounds revealed that all of them were significantly more abundant in the skin than in the flesh. In addition, the major compounds within each category showed a variation pattern similar to that previously described for total polyphenol content during postharvest ripening.

The components making the greatest contribution to total polyphenol content in the apple skin were those of the flavanol category (Figure 3). The predominant compounds were procyanidin B2 (Figure 3A) and epicatechin (Figure 3B), both with high levels in the green fruit (day 0), followed by catechin (Figure 3C), whose content was approximately seven-fold lower. The three compounds decreased markedly in content at day 7 and subsequently increased their levels as ripening progressed. Procyanidin B2 recovered levels similar to those observed in the green fruit as early as day 14, whereas epicatechin and catechin showed more gradual increases, reaching their maximum values in the ripe apple (day 35). In the flesh, procyanidin B2 (Figure 3D) was also the predominant flavanol, followed by epicatechin (Figure 3E) and, to a lesser extent, catechin (Figure 3F), whose levels were approximately six-fold lower than those of epicatechin. In this tissue, the three compounds decreased significantly at day 7 and remained at low levels throughout the postharvest ripening period.

ACC treatment alone barely modified the profile described for the control group, with only a stimulatory effect observed on procyanidin B2 in the skin at day 35 and in the flesh at days 7 and 35. By contrast, treatments with melatonin alone generally exerted a stimulatory effect on flavanol content relative to the control at each study time. In the skin, the higher MEL concentrations (50 and 500 µM) significantly increased the content of procyanidin B2, epicatechin and catechin at days 7 and 14, although this effect was not maintained at day 35. In the flesh, all melatonin concentrations produced significant increases at day 14 for the three compounds, whereas the higher doses significantly stimulated their levels at day 7 and, in the case of procyanidin B2 and catechin, also at day 35. In addition, the lowest MEL concentration also significantly increased catechin at this last time. The MEL + ACC combination likewise showed a stimulatory effect. In the skin, the high MEL doses in combination with ACC increased the content of the three flavanols at the onset of ripening and that of procyanidin B2 at day 35. In the flesh, this effect was observed for procyanidin B2 throughout the period studied, for epicatechin at days 7 and 14, and for catechin only on day 7.

**Figure 3:**
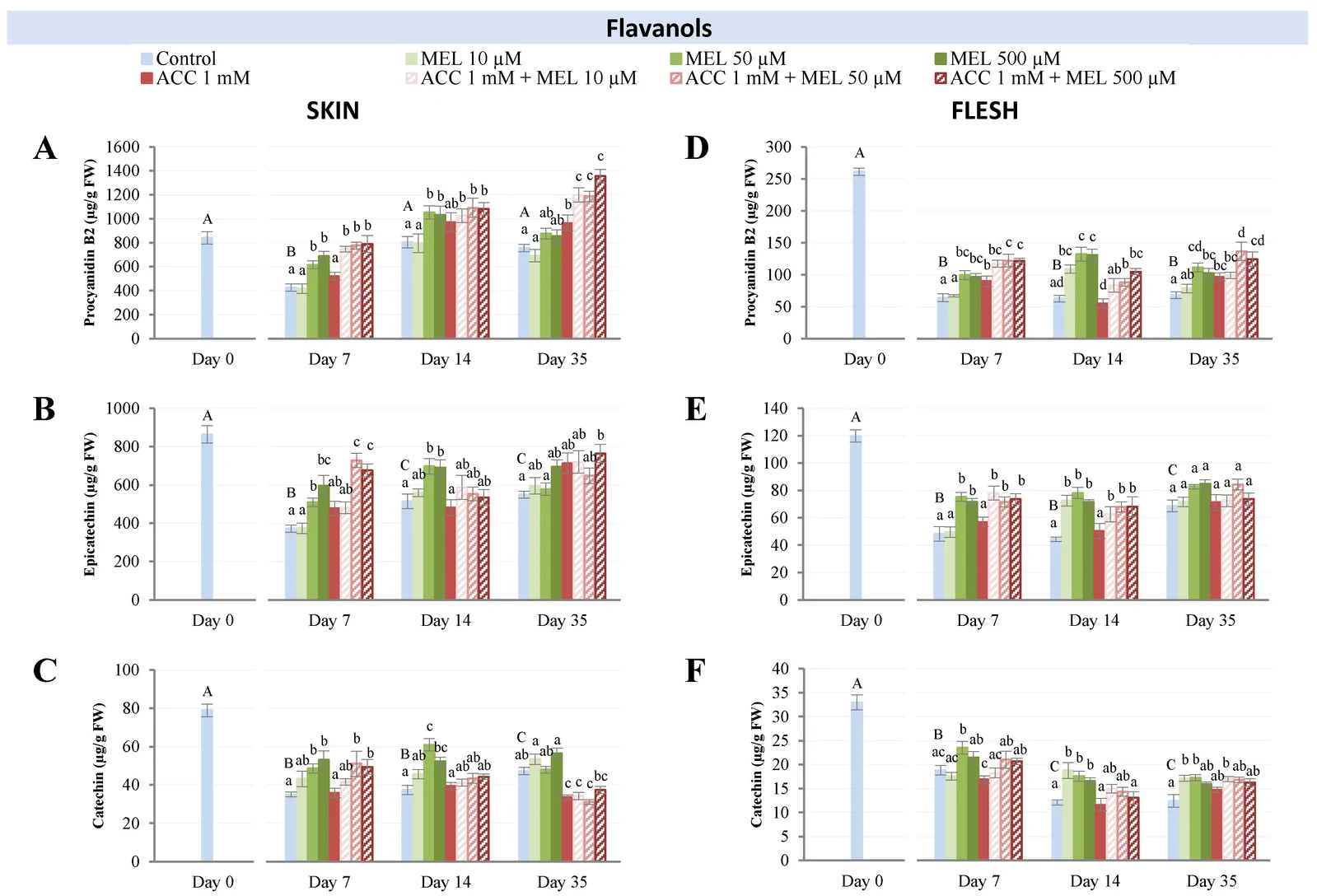
Polyphenolic profile of the flavanol group (µg/g FW) in skin (**A**–**C**) and flesh (**D**–**F**) samples of ‘Reineta’ apples treated with melatonin (MEL), ACC, and their combination (MEL + ACC) during the postharvest period. Identified compounds: (**A**,**D**) procyanidin B2, (**B**,**E**) epicatechin, and (**C**,**F**) catechin. Statistics: different capital letters indicate significant differences over time for the control group, *p* < 0.05; different lowercase letters indicate significant differences between treatment groups within the same time, *p* < 0.05.

In the category of simple phenolic acids (Figure 4), chlorogenic acid was the predominant compound in both the skin and flesh of the green fruit (Figure 4A,E), whereas the remaining components showed considerably lower levels. With the exception of *o*-coumaric acid, whose maximum content in the skin was observed at day 14, the other compounds showed a similar pattern in both tissues, characterized by a significant decrease at the onset of ripening (day 7) and levels that remained relatively stable until the end of the process. ACC barely altered this profile, although it produced some specific variations relative to the control, such as increases in chlorogenic acid in the flesh at days 7 and 35 (Figure 4E) and in *o*-coumaric acid in both tissues at day 35 (Figure 4E,H). Likewise, decreases were observed in the contents of *o*-coumaric, caffeic and *p*-coumaric acids in the skin at day 7 (Figure 4B–D), a pattern also observed in the flesh for caffeic and *p*-coumaric acids (Figure 4G,H).

Melatonin applied alone produced a marked stimulatory effect on chlorogenic acid relative to the control at each time (Figure 4A,B). In the skin, the three concentrations tested significantly increased its content at days 7, 14 and 35, maintaining high and relatively constant levels throughout the ripening period. In the flesh, where this compound constitutes the main phenolic acid, MEL also induced highly significant increases at days 7, 14 and 35, reaching at all sampling times levels similar to those of the green fruit. The MEL + ACC combination showed effects comparable to those obtained with MEL alone in both tissues and throughout the postharvest period. Moreover, the higher MEL concentrations in combination with ACC produced levels significantly higher than those obtained with ACC alone, at days 7 and 35 in the skin and at day 7 in the flesh.

For the remaining phenolic acids, whose contribution was minor, melatonin and its combination with ACC generally showed stimulatory effects relative to the control, although without a uniform temporal pattern. Thus, the higher MEL concentrations increased the content of caffeic acid in the skin at days 14 and 35 and in the flesh at days 7 and 14 (Figure 4C,G). Similar effects were observed with MEL + ACC, even reaching levels significantly higher than those obtained with ACC alone at days 7 and 14 in the case of the skin. Likewise, the higher MEL doses increased *o*-coumaric acid in the skin at days 7 and 35 (vs. control at each time), with a stimulatory effect also observed at day 7 with the combination of all melatonin doses with ACC (Figure 4B). In the case of *p*-coumaric acid, the higher melatonin concentrations increased its content in the flesh at days 7 and 35, an effect also observed with the MEL + ACC combination (Figure 4H). Although other stimulatory effects were detected on various compounds and treatment times, these did not reach statistical significance.

Phloridzin was the only compound belonging to the dihydrochalcone category detected in both skin and flesh (Figure 5A,B). In the freshly harvested apple, its levels were approximately five-fold higher in the skin. In both tissues and in the absence of treatment, its content decreased significantly at day 7 and these levels remained relatively constant until the ripe state of the fruit. ACC alone produced no significant changes in the content of this compound relative to the control. However, melatonin applied alone exerted a stimulatory effect in the skin at day 7 for the three doses tested and at day 35 for the higher concentrations (vs. control at each time). In the flesh, this effect only reached statistical significance with the higher doses at day 35, although this trend was also observed at days 7 and 14. The MEL + ACC combination significantly increased phloridzin content in the flesh at days 14 and 35 and in the skin at days 7 and 35.

In the flavonol category, avicularin and rutin were the predominant compounds. In the skin, both reached their maximum levels at day 14 (Figure 6A,B), whereas in the flesh, their contents were considerably lower (Figure 6D,E). Quercetin showed low and variable levels in both tissues (Figure 6C,F). ACC treatment alone barely modified the content of these compounds relative to the control at each time point. By contrast, melatonin generally produced stimulatory effects. In the skin, the MEL 50 and 500 µM doses markedly increased rutin content at days 14 and 35 (Figure 6A), whereas avicularin increased significantly at day 7 with the three melatonin concentrations and at day 14 only with the two higher ones (Figure 6B). The MEL + ACC combination also increased rutin content at days 7 and 35 and avicularin content at day 7, relative to ACC treatment alone. As for quercetin, the three melatonin doses significantly increased its content at day 7 and, for the higher doses, at day 35 (Figure 6C). In the flesh, the three MEL concentrations increased quercetin content throughout ripening, in both the absence and the presence of ACC (Figure 6F). Although increases were also observed in the contents of avicularin and rutin, these did not reach statistical significance in this tissue (Figure 6D,E).

## 3. Discussion

### 3.1. Postharvest Ripening and Fruit Quality Parameters: Effect of Melatonin and ACC

The evolution of the main quality attributes of ‘Reineta’ apple over the 35 days of postharvest storage followed, in the control group, the pattern characteristic of a climacteric fruit of *Malus domestica*: a progressive increase in soluble sugar content, a sustained loss of firmness, a chromatic transition from the initial green to a warm background color with the reddish striping characteristic of the cultivar, and a marked increase in ethylene production between days 14 and 35 (Table 1). This pattern is consistent with the ascending and post-climacteric phases of the autocatalytic burst previously characterized in this cultivar; in apple, this burst is driven by the induction of MdACS1 and MdACO1 [59].

Exogenous melatonin application accelerated the progress of ripening. All doses tested significantly increased ethylene production relative to the control at day 7, when climacteric biosynthesis is still in its initial ascending segment, and maintained this stimulatory effect at days 14 and 35. This promoting effect on ethylene biosynthesis is consistent with previous observations in ‘Reineta’ and other cultivars such as ‘Golden’, ‘Blanquina’ and ‘Rabiosa’ [59]; in ‘Golden’, melatonin was shown to activate the transcription of MdACS1 and MdACO1 in the skin, which acts as the initiating tissue of the climacteric burst. Recent genetic evidence in tomato reinforces this pro-climacteric role, showing that the balance between melatonin biosynthesis and catabolism modulates the rate of ripening through the overexpression of ethylene biosynthetic genes and of components of the ethylene signaling cascade [49]. These data reinforce the notion that melatonin operates, in climacteric fruits such as tomatoes and apples, as a transcriptional activator of ethylene biosynthesis and signaling.

This response contrasts, however, with the inhibition of ethylene described in ‘Fuji’ [67] or in other species such as pear [51], banana [50] and peach [53]. This divergence may reflect a cultivar–physiological stage–dose interaction, in which melatonin would act as an accelerator in fruit harvested at advanced pre-climacteric stages or with a still-latent ripening program, and as a brake when the fruit is in the full climacteric phase or already senescent. It should also be borne in mind that the applied concentration, the route of administration (immersion vs. spraying) and the storage temperature are additional factors that may condition the direction of the effect [68].

Despite the promotion of ethylene observed, melatonin significantly delayed softening, especially at day 35, where treated apples retained 16–27% higher firmness than the control. This uncoupling between ethylene stimulation and firmness retention is one of the most relevant findings of the present study and is particularly striking when contrasted with the behavior expected in apples treated with ethylene precursors or releasers. Thus, in the cultivar ‘Honeycrisp’, the application of ACC or ethephon induces an accelerated loss of firmness linked to the overexpression of *MdPG1* (polygalacturonase), whereas inhibition of ethylene biosynthesis with AVG preserves firmness [69]. In our study, ACC advanced ethylene production but did not significantly modify firmness, whereas the MEL + ACC combination maintained values close to those of melatonin alone, despite an even greater ethylene production. This pattern suggests that melatonin acts on targets additional to ethylene, probably linked to cell-wall integrity, membrane stability and the control of oxidative stress associated with senescence. The intracellular accumulation of reactive oxygen species (ROS) constitutes a central mechanism driving postharvest softening, insofar as the hydroxyl radical generated directly attacks cell-wall polysaccharides and promotes dissolution of the middle lamella [70]. The antioxidant action of melatonin, together with its capacity to induce antioxidant enzymes (SOD, CAT, APX, GR) in apples, peaches and other climacteric fruits [56,67,71], would thus provide a redox protection that would mitigate the oxidative component of softening even under an amplified ethylene signal. However, it should be noted that none of these mechanisms—cell-wall integrity, membrane stability or control of the redox state—were directly measured in the present study. Their formulation therefore constitutes a mechanistic hypothesis consistent with the available literature, but one that requires specific experimental verification in this cultivar through the determination of cell-wall enzyme activity and oxidative stress markers.

Soluble sugar content, expressed as °Brix, increased significantly under melatonin from day 14 onward, with the highest values corresponding to MEL 50 µM at day 35 (Table 1). This result agrees with that described in ‘Reineta’ and ‘Golden’ [59], ‘Zaosu’ pear [72], tomato [21] and peach [52], where melatonin stimulated the activity of enzymes involved in sucrose metabolism, such as sucrose phosphate synthase and sucrose synthase [73]. Consistent with this, in the ‘Honeycrisp’ apple, the ACC and ethephon treatments increase °Brix through accelerated starch degradation, a typically ethylene-dependent effect [69]. In our work, melatonin produced a more potent effect than ACC on this parameter, suggesting that, in addition to inducing the climacteric, melatonin incorporates additional effects on sucrose metabolism.

The acceleration of color observed with all melatonin concentrations, particularly at the lower doses, is consistent with faster chlorophyll degradation and with the ethylene-dependent accumulation of carotenoids and anthocyanins, processes that in other fruits are modulated at the transcriptional level by melatonin [5,45,74]. In pears, melatonin has been shown to induce an ERF–MYB regulatory module that simultaneously activates anthocyanin biosynthesis and the vacuolar transport of these pigments, amplifying fruit coloration [75]. An analogous mechanism could operate in apples, where *MdMYB10* is the key regulator of red pigmentation and functional orthologs of the genes involved have been identified [69]. In this context, the fact that melatonin surpasses ACC in inducing the color change at day 35 suggests that its effect is not limited to enhancing the ethylene-dependent pathway but could be activating, in parallel, an additional component through ERF–MYB-type cascades capable of reinforcing both anthocyanin biosynthesis and its transport and accumulation in the vacuole.

ACC treatment partially reproduced the effects of melatonin. Although it advanced the onset of ethylene production and accelerated the color change, it did not significantly modify firmness or sugar content relative to the control. These results indicate that ethylene alone does not account for the full set of responses induced by melatonin, and support the hypothesis that the indoleamine could act through a dual pathway: an ethylene-dependent one, linked to climacteric induction, and an ethylene-independent one, linked to its antioxidant capacity and to the activation of secondary metabolism. The ACC+MEL combination showed a complex interaction pattern. On ethylene production, the effect was clearly synergistic, with increases 2- to 3-fold greater than the individual treatments at day 14, suggesting that melatonin enhances both precursor availability and the activity of the biosynthetic machinery (ACS and ACO). The coarse sampling of the present work did not resolve the climacteric maximum, but a higher-resolution study on the same cultivar placed the peak at around day 21 and showed that melatonin applied alone was the only treatment able to delay it, by about four days [59]. This indicates that melatonin, on its own, not only amplifies the magnitude of the climacteric burst but also affects its temporal distribution, prolonging the active phase of ethylene production and broadening the physiological ripening window. By contrast, when combined with exogenous ACC, the response could be limited by ACO activity and by the massive availability of the precursor itself. This could saturate the control point regulated by melatonin, such that the initial kinetics of the ethylene peak would be that observed for untreated fruit.

With respect to firmness and sugars, however, the behavior of the MEL + ACC combination was equivalent to that of melatonin applied alone, with the highest firmness and °Brix values reached at day 35 with the intermediate and high doses. The retention of firmness in the face of a quantitatively amplified ethylene stimulus reinforces the conclusion that the role of melatonin in postharvest quality is not channeled solely through ethylene but includes an autonomous protective component acting on the somatic tissues of the fruit.

### 3.2. Total Phenolic and Flavonoid Contents in Skin and Flesh

The basal distribution of phenolic content was markedly asymmetric between the two tissues analyzed, with values approximately 2–3-fold higher in the skin for total phenolics and three-fold for total flavonoids, in agreement with that described for *M. domestica* and other pome and stone fruits [12,76,77]. This tissue compartmentalization is consistent with the role of the skin as the first barrier against biotic and abiotic stressors and as the preferential site of light-dependent flavonoid biosynthesis, regulated by transcription factors such as *MdMYB10* and *MdMYB1*, whose expression is strongly modulated by temperature and irradiance [78].

In the absence of treatment, total phenolics decreased significantly at the onset of ripening (day 7) in both skin and flesh and partially recovered in the following days without reaching the levels of the green fruit (Figure 1). This pattern has also been described in other apple varieties during on-tree and postharvest ripening [15,16,79] and could indicate the existence of an initial period of phenolic mobilization and oxidation, followed by reactivation of the phenylpropanoid biosynthetic pathway as ripening progresses. However, total flavonoids showed opposite tissue dynamics: a decline and recovery in the skin and a transient maximum at day 7 followed by a gradual decline in the flesh (Figure 2). This divergence is consistent with the different distribution of flavonoid groups in the two tissues—flavanols, flavonols and dihydrochalcones predominating in the skin; flavanols and hydroxycinnamic derivatives in the flesh—and with the greater sensitivity of the flesh to oxidative processes possibly mediated by the enzyme polyphenol oxidase after harvest.

Three-way ANOVA confirmed a significant effect of melatonin on total phenolic and total flavonoid contents in both tissues (*p* < 0.001; Table 2). Melatonin maintained total phenolics at levels close to those of the green fruit, with significant increases relative to the control at day 7 (all doses) and at day 14 (50 and 500 µM). In the flesh, this effect was significant for the highest doses at days 7 and 14 (Figure 1). At day 35, the absence of significant effects could reflect not a loss of the melatonin effect but rather that the control had reached polyphenol values similar to those of the treated groups, which is to be expected with the climacteric induction of secondary metabolism. For total flavonoids, a significant effect of melatonin was found that was independent of time (with no Time × MEL interaction in either tissue; Table 2), suggesting the existence of a basal and sustained activation of the flavonoid biosynthetic pathway, possibly mediated by transcriptional induction of the key enzymes (PAL, CHS, CHI, F3H and FLS), as demonstrated in tomato [45], pear [74], grape [5,80], guava [81] and litchi [71], where melatonin treatment preserved high contents of phenolics, flavonoids and anthocyanins during storage through simultaneous inhibition of polyphenol oxidase and activation of the phenylpropanoid biosynthetic pathway.

ACC acted with a profile distinct from that of melatonin: it did not significantly modify total phenolic content in the skin but did so in the flesh, and it stimulated total flavonoids in the skin at days 7 and 14, with no detectable effect in the flesh (Figure 1 and Figure 2). These data suggest that ethylene by itself partially induces the biosynthetic pathway of these compounds, as also occurs with the ethylene-dependent regulation of biosynthetic genes described in grapes [5] and other climacteric fruits [66], but its capacity to maintain phenolic content is clearly inferior to that of melatonin. For its part, the MEL + ACC combination produced total-phenolic levels in the skin similar to those of melatonin applied alone at day 7, with a trend toward non-significant increases at later times. In the flesh, the MEL × ACC interaction was significant for total phenolics (*p* = 0.021; Table 2), although without a clear pattern of potentiation. In the case of total flavonoids, the combined treatment increased levels in the skin relative to the control and, at day 7, also relative to ACC, but the stimulatory effect observed for melatonin on flesh flavonoids was lost when combined with ACC at day 35. Our data do not allow the cause of this interaction to be established. One possibility is that part of the flavanol pool was converted into products not quantified by the analytical method employed: flavanol oligomerization yields proanthocyanidins of higher degrees of polymerization, while polyphenol oxidase-mediated oxidation generates quinones that subsequently polymerize into high-molecular-weight compounds, none of which are detected by the HPLC-DAD determination of individual compounds used here. Whether the combined treatment actually promotes such conversions remains to be established and would require the specific quantification of this high-molecular-weight fraction.

### 3.3. Polyphenolic Groups: Relative Contribution and Tissue Redistribution During Ripening

The eleven phenolic compounds quantified confirmed a conserved tissue organization in *M. domestica*: in the skin, flavanols were clearly dominant, followed by phenolic acids, dihydrochalcones and flavonols; in the flesh, flavanols and phenolic acids were likewise predominant, whereas dihydrochalcones and flavonols were very minor (Table 3 and Table 4). This chemical architecture matches that described for various apple varieties [12,13,82,83,84] and reflects the functional specialization of each tissue: the skin concentrates flavonols, anthocyanins and dihydrochalcones associated with photoprotection and defense, whereas the flesh, less exposed, maintains a pool of flavanols and hydroxycinnamic acids, chiefly chlorogenic acid, linked to internal redox regulation [83,84].

A notable result is the inversion of the predominant group in the flesh: phenolic acids overtook flavanols by day 35 (Table 4). This reorganization of polyphenolic categories is consistent with the differential dynamics observed for the individual flavanols—a sharp drop at day 7 without subsequent recovery in the flesh—and with the greater relative stability of chlorogenic acid, which in the flesh acts as the predominant antioxidant reservoir. By contrast, the skin maintained its initial pattern throughout ripening, indicating a greater capacity to retain the flavanol pattern in the epidermal tissue, possibly associated with its binding to cell-wall structures and accumulation in the vacuole.

The treatments tested did not alter the relative proportions among polyphenolic groups but did significantly modify the absolute content. The generalized decrease in polyphenols at day 7 relative to the green fruit was more pronounced in the ACC treatments and with the lowest melatonin dose but was attenuated with the intermediate and high melatonin doses and with the MEL + ACC combination, which maintained levels significantly higher than the control in both tissues (Table 3 and Table 4). This attenuation of the postharvest decline is one of the most reproducible effects of melatonin in climacteric and non-climacteric fruit [60,85,86] and is interpreted both as inhibition of the oxidative degradation of phenolics (PPO inhibition) and as activation of de novo biosynthesis through the phenylpropanoid pathway.

During advanced ripening, melatonin restored the skin’s total polyphenols to green-fruit levels, whereas in the flesh it raised the content without reaching the initial values. This asymmetry reflects the greater biosynthetic capacity of the epidermal tissue and the partially irreversible nature of the phenolic decline observed at the start of the process in the flesh. In this tissue, the MEL + ACC combination significantly exceeded ACC only at day 35 and exclusively with MEL 50 µM. This suggests that the synergistic effect between the two compounds is limited to a range of melatonin concentrations, losing added value outside it.

This tissue-specific response—restoration of polyphenolic content to green-fruit levels in the skin but not in the flesh—may result from several non-exclusive factors. First, the skin has a markedly higher biosynthetic capacity for phenolic compounds, as it concentrates the enzymatic machinery of the phenylpropanoid and flavonoid pathways and acts as the main site of synthesis and storage of flavonols, anthocyanins and dihydrochalcones [12,77]. Second, flavonoid biosynthesis in the epidermis is strongly regulated by light, as noted above, so that the surface tissue has an additional activation route that the non-exposed flesh lacks. Third, the phenolic decline observed in the flesh at the onset of ripening was more pronounced and apparently irreversible, suggesting a lower capacity of this tissue to reactivate de novo synthesis once senescence is advanced, in contrast to the effective reactivation shown by the skin. Together, these factors would account for the more intense and reproducible stimulatory effect of melatonin on phenolic metabolism in the skin than in the flesh.

From the standpoint of fruit quality, this asymmetry has relevant implications. The skin concentrates most of the antioxidant potential of the apple and is, moreover, the tissue that determines fruit appearance and initial consumer acceptance; a preferential phenolic enrichment in this tissue, together with the intensification of the characteristic coloration of the cultivar, would contribute directly both to the nutritional and functional value of the fruit and to its commercial appeal. The smaller effect in the flesh is consistent with the role of this tissue as a reservoir of hydroxycinnamic acids—chiefly chlorogenic acid—linked to internal redox regulation rather than to surface defense.

### 3.4. Effect of the Treatments on Individual Polyphenolic Compounds

#### 3.4.1. Flavanols (Epicatechin, Procyanidin B2, Catechin)

The three quantified flavanols showed a temporal pattern consistent with the group’s total content: high levels in the green fruit, a pronounced decline at day 7 and progressive recovery in the skin, with maxima at day 35 for epicatechin and catechin, and at day 14 for procyanidin B2.

The temporal pattern of the three quantified flavanols (Figure 3) agrees with that described by Mayr et al. [76] in ‘Golden Delicious’, in which flavanols evolve from simple forms toward more complex structures as the fruit ripens, accompanied by a general decline in their content. Awad et al. [16] and Renard et al. [18] corroborated this same pattern, consolidating it as a characteristic feature of phenolic development in apples.

ACC barely modified the basal profile, whereas melatonin exerted a clear stimulatory effect on the three flavanols, more marked in the skin at the earlier sampling times and extending in the flesh to day 35 for procyanidin B2 and catechin (Figure 3). The MEL + ACC combination maintained this stimulatory profile, particularly on procyanidin B2, whose flesh content exceeded that of ACC throughout the period. The molecular basis of this response has recently been elucidated in grape seeds, where ethylene has been shown to raise the endogenous melatonin content, which in turn activates MYB14, a key regulator triggering the early and late steps of the phenylpropanoid pathway, including the synthesis of flavanols and proanthocyanidins [87]. This would explain why MEL + ACC maintains procyanidin B2 levels throughout the postharvest period and why the effect is clearer on flavanols, as end products of the pathway, than on other intermediates. The preference of melatonin for sustaining the procyanidin reservoir is also of practical interest, since these compounds have been associated with fruit firmness, presumably through their interaction with cell-wall polysaccharides. As regards astringency, it should be noted that this attribute depends largely on the degree of polymerization of procyanidins; since the high-molecular-weight oligomeric fraction was not quantified in this work, the net effect of melatonin on this sensory attribute cannot be inferred from the present data and would require specific organoleptic evaluation.

#### 3.4.2. Simple Phenolic Acids (Chlorogenic, Caffeic, P-Coumaric, O-Coumaric)

Chlorogenic acid was the major compound of the group in both skin and flesh (Figure 4A,E). Moreover, in the flesh, it is the most abundant individual polyphenol in the ripe fruit. This result reflects that it is one of the most relevant compounds in apple physiology, both as the preferential substrate of polyphenol oxidase and for its antioxidant function within the cell [13,83,88]. Melatonin, at all doses, induced a highly significant increase in chlorogenic acid in both tissues, with levels close to those of the green fruit throughout the postharvest period, an effect maintained in the MEL + ACC combination and surpassing, at the higher doses, that of ACC alone. The sustained activation of flux toward chlorogenic acid suggests that melatonin acts specifically on the early steps of the phenylpropanoid pathway, as also described in grape, guava and litchi [71,81,87], as well as on hydroxycinnamoyl-CoA quinate transferase (HQT), a key enzyme in the synthesis of the compound [62].

Caffeic, *o*-coumaric and *p*-coumaric acids showed lower abundance and more variable dynamics (Figure 4B–D,F–H), with a generally stimulatory effect of melatonin and of the MEL + ACC combination, without a homogeneous temporal pattern. Of note is the increase in *o*-coumaric acid in the skin at day 14 (Figure 4B), which fits with a transient activation of the final steps of the hydroxycinnamic pathway in the period preceding the climacteric peak, although this is a pattern still poorly documented in apple.

#### 3.4.3. Dihydrochalcone Derivatives (Phloridzin)

Phloridzin, the only quantified representative of the group, showed levels approximately five-fold higher in the skin than in the flesh (Figure 5A,B), in line with its preferential distribution described in *M. domestica* [12,77]. Melatonin, and more markedly the MEL + ACC combination, significantly stimulated its content in both tissues at the later sampling times (Figure 5), pointing to a specific stimulatory effect on phloridzin synthesis, probably through activation of chalcone synthase and of the enzymes responsible for its glycosylation. This mechanism has already been documented in melatonin-treated fruit [45,74,87].

#### 3.4.4. Flavonols (Rutin, Avicularin, Quercetin)

Avicularin and rutin, both glycosidic derivatives of quercetin, were the major flavonols in the skin, where melatonin—and its combination with ACC—significantly increased their content relative to the control and to ACC (Figure 6A,B). In the flesh, where these glycosides were marginal, the response of quercetin stands out (Figure 6F): its content was significantly stimulated by all three melatonin doses at all sampling times, with or without ACC. The greater effect on quercetin than on its glycosides in the flesh may reflect that the final steps of flavonol-skeleton synthesis are especially sensitive to melatonin and that, during late ripening, a redistribution between free and conjugated forms of this compound may occur [74,87].

### 3.5. Integration: Melatonin as a Promoter of Fruit Quality Despite Ethylene Stimulation

Taken together, the results outline a physiological scenario of apparent paradox: melatonin accelerated typical markers of climacteric ripening—ethylene production, sugar accumulation, color change—and yet delayed softening and sustained high levels of phenolics, flavonoids and individual polyphenols, particularly in the skin. This combination defines a favorable postharvest quality profile, characterized by a fruit that is more organoleptically ripe, more colored and sweeter, yet firmer and with a reinforced antioxidant pool.

The simplest interpretation of this duality is that melatonin acts not through a single pathway but simultaneously on two partially independent axes. On the one hand, it accelerates ripening: it activates *MdACS1* and *MdACO1*, increases ethylene availability and, with it, reproduces much of the effect of ACC on color, sugars and overall ripening [59]. A recent study in tomatoes reinforces this idea by demonstrating that the balance between production and degradation of endogenous melatonin directly controls the rate of ripening through the ethylene cascade [49]. The second axis is protective in nature. In this case, melatonin, in an ethylene-independent manner, curbs postharvest deterioration: it acts as a direct antioxidant, induces the phenylpropanoid pathway and activates the main defense enzymes—SOD, CAT, APX and POD—keeping under control the accumulation of ROS, whose excess is one of the main triggers of softening and postharvest quality loss through cell-wall degradation and lipid peroxidation [60,68,70,71]. The two axes need not act in parallel, since ethylene signaling and melatonin may converge on the same transcriptional modules regulating polyphenol synthesis and fruit pigmentation, as illustrated by the ERF–MYB cascades described above in grape and pear. Applied to ‘Reineta’ apple, this model suggests that melatonin promotes ripening and protects the fruit through a shared regulatory framework rather than through mutually exclusive pathways, which would account for the fact that ACC alone reproduced the ethylene-related responses without modifying firmness or phenolic content.

The accumulation of chlorogenic acid, procyanidin B2 and rutin—polyphenols with strong antioxidant activity and the capacity to interact with the cell wall (Figure 3, Figure 4 and Figure 6)—coincided in time with the retention of firmness. It must be stressed, however, that the present data do not allow a direct causal relationship to be established between phenolic enrichment and firmness retention. This correlation nonetheless suggests a possible contribution of these compounds to the structural stability of the tissue, plausibly through their interaction with pectic polysaccharides and the consequent limitation of polygalacturonase and expansin activity, enzymes whose ethylene-dependent induction is the most direct cause of softening in apples [69].

In the flesh, however, the response to the combined treatment was more heterogeneous, with synergistic effects on some individual compounds (procyanidin B2, chlorogenic acid, phloridzin, quercetin) at particular melatonin concentrations, and loss of the melatonin-induced increase in total flavonoids at day 35, indicating that the melatonin dose requires fine adjustment when combined with ethylene precursors.

Two further considerations concern the determinations performed. First, the sampling at 0, 7, 14 and 35 days does not resolve the fine kinetics of the climacteric burst, whose temporal structure in ‘Reineta’ and response to the treatments were characterized with greater resolution elsewhere [59] and are discussed above; accordingly, the ethylene values at day 14 with MEL + ACC most likely correspond to the expanded ascending phase, and those at day 35 to the incipient post-climacteric senescence, in which melatonin continues to sustain firmness, organoleptic quality and phenolic content. Second, the quantification is limited to eleven polyphenolic compounds and to the total content estimated by colorimetric methods, so that the dynamics of anthocyanins—relevant to the color change of ‘Reineta’—and of oligomeric procyanidins with a high degree of polymerization fall outside the scope of the analysis.

The present findings refer to ripening at ambient temperature and cannot be directly extrapolated to the low-temperature regimes (0–4 °C), often under controlled atmosphere, of commercial apple storage. The dual model proposed here nonetheless allows a tentative projection: the ethylene-dependent axis, being temperature-sensitive, would foreseeably be attenuated under refrigeration, since cold suppresses the climacteric, whereas the protective, ethylene-independent axis—antioxidant capacity, firmness and phenolic retention—might operate more transversally, albeit with slower kinetics. These remain hypotheses, and the efficacy and kinetics of melatonin under refrigerated or controlled-atmosphere storage would require specific evaluation.

Future studies should incorporate determinations specifically aimed at discriminating the pathways involved in this uncoupling. On the one hand, measuring the activity of the main cell-wall-degrading enzymes—polygalacturonase (PG), pectin methylesterase (PME) and β-galactosidase—would establish whether the firmness retained under melatonin treatment is associated with reduced depolymerization of pectic polysaccharides. On the other, the quantification of hydrogen peroxide (H_2_O_2_) and malondialdehyde (MDA), together with the activity of the antioxidant enzymes SOD, CAT and APX, would determine to what extent the protective effect operates through the attenuation of the oxidative stress and lipid peroxidation associated with senescence [70]. The quantification of anthocyanins and the analysis of the expression of structural and regulatory genes of the phenylpropanoid pathway would complete the characterization of the mechanism underlying the uncoupling between ethylene and firmness.

The dual nature of melatonin action could help to explain the apparent heterogeneity of its effects among species: whereas its influence on ethylene signaling, and therefore on the direction of the ripening process, appears to depend strongly on the species and the physiological stage, its protective component, antioxidant and ethylene-independent in nature, could operate more transversally, which would be consistent with the improvement of postharvest quality described in climacteric and non-climacteric fruits of very diverse behavior.

From an applied standpoint, melatonin can be delivered both as a preharvest spray on the tree, which in previous work on these cultivars improved final fruit quality [58], and as the postharvest immersion used in the present study. The choice between the two would depend on the technological objective pursued—modulation of ripening in the field versus preservation of quality after harvest.

Overall, this study identifies melatonin as a physiological modulator of postharvest ripening in ‘Reineta’ apples, capable of simultaneously enhancing the climacteric transition and the nutritional and mechanical quality of the fruit. Under the ambient-temperature ripening conditions assayed here, melatonin maintained higher firmness, intensified the characteristic coloration of the cultivar and enriched the polyphenolic profile, particularly in the skin. The uncoupling between ethylene stimulation and firmness retention revealed by these results indicates that the action of melatonin on fruit quality is not confined to the ethylene pathway and identifies this indoleamine as a promising tool for modulating apple ripening after harvest.

## 4. Materials and Methods

### 4.1. Plant Material and Experimental Design

‘Reineta’ apples were harvested from the certified organic orchard “Torres de Moreda” (Callobre, A Estrada, Spain) in October 2024, 135 days after anthesis. The harvest date was established on the basis of the fruit growth curves previously determined for this cultivar [58], corresponding to the point at which fruit size and weight reached a plateau, indicating that fruit expansion was complete. At harvest, fruit showed a mean weight of 81.90 ± 2.36 g, an equatorial perimeter of 17.33 ± 0.25 cm, a firmness of 19.95 ± 0.64 kg/cm^2^, a total soluble solids content of 10.35 ± 0.15 °Brix, and a fully green background skin color (Table 1, day 0). The pre-climacteric condition of the fruit was confirmed by ethylene production remaining below the detection limit of the method both at harvest and at day 7 of storage in control fruit (Table 1).

Once in the laboratory, apples were carefully washed and visually inspected. Fruit showing mechanical injuries, bruises, disease symptoms or any other visible defect was discarded. From the remaining fruit, a uniform batch was selected by visual assessment of homogeneity in weight, equatorial diameter and background skin color, in order to minimize initial variability among experimental groups.

Fruit was kept in controlled-environment chambers at 22 ± 2 °C, 70% relative humidity and a 12 h light/12 h dark photoperiod throughout the 35-day ripening period, thus reproducing a natural day/night cycle. These conditions, identical to those used in previous work on these cultivars [59], were selected to allow the ripening program to progress actively within the experimental period, since at commercial refrigeration temperatures the climacteric transition is largely suppressed, and to ensure that temperature, humidity and photoperiod remained constant and identical across all treatment groups, so that differences between groups could be attributed to the treatments themselves. It should be noted that these conditions correspond to postharvest ripening at ambient temperature and not to commercial cold storage.

The experiment comprised eight treatment groups: control (vehicle solution), melatonin (MEL) at 10, 50 and 500 µM; 1-aminocyclopropane-1-carboxylic acid (ACC) at 1 mM; and the three combinations of ACC with each melatonin concentration (MEL + ACC). Melatonin, as the factor under study, was assayed at three concentrations to explore its range of action, whereas ACC was included at a single, saturating concentration (1 mM) as a reference treatment for ethylene induction rather than as a factor to be dose-varied: its purpose was to provide a stable ethylene-related reference against which the ethylene-dependent and ethylene-independent effects of melatonin could be discriminated, so that any additional effect observed in the combined treatments could be attributed to melatonin and not to a variation in the ethylene stimulus. Fruits were randomly assigned to the experimental groups and numbered, and baseline data on size, weight and color were recorded.

Melatonin was first dissolved in absolute ethanol and subsequently diluted in distilled water to the required concentrations, yielding a final ethanol concentration of 5% (*v*/*v*) in all treatment solutions, including the melatonin–ACC combination. Control fruit was immersed in the same vehicle solution (5% ethanol, *v*/*v*, in distilled water) without melatonin, so that any effect attributable to the solvent was accounted for. ACC was dissolved in the same vehicle solution at 1 mM, either alone or combined with melatonin. All fruit belonging to a given group was immersed together in the corresponding solution for 2 h. The treatment concentrations and application times were selected based on previous studies on climacteric fruits, in which these ranges elicited measurable effects on ethylene biosynthesis and fruit ripening [21,50,59,89]. Apples were then left to dry for 12 h at room temperature and kept for ripening for 35 days under the environmental conditions described above.

Based on previous results obtained for this cultivar regarding the evolution of ethylene production [59], four sampling events were scheduled: (i) day 0, control group only, corresponding to freshly harvested fruit (hereafter referred to as green fruit), which had completed growth and reached physiological maturity but had not yet initiated ripening, with a fully green background skin color, maximal firmness and ethylene production below the detection limit of the method; (ii) day 7, all groups (before the onset of ethylene production in control fruit); (iii) day 14, all groups (rise in climacteric ethylene production); and (iv) day 35, all groups, final ethylene production (fruit fully ripe, prior to senescence).

For each treatment group and sampling time, 10 fruits were used, and all determinations were carried out on each fruit individually (*n* = 10). The same 10 fruits per group and sampling time were used for all the determinations reported in this study, which were performed sequentially on each fruit: first the non-destructive measurements (skin color and ethylene production), followed by the destructive ones (firmness and total soluble solids), after which skin and flesh samples were taken from each fruit under sterile conditions, immediately frozen in liquid nitrogen and stored at −80 °C until analysis. Skin and flesh from each individual fruit were extracted and analyzed separately, without pooling. All data are expressed as mean ± standard error (SE) of 10 individual fruits per treatment group and sampling time.

### 4.2. Determination of Apple Ripening Parameters

#### 4.2.1. Fruit Quality Measurements

Fruit firmness was assessed using a texture analyzer (Texture Analyzer, DT120, Mecmesin Ltd., Slinfold, UK) equipped with a 6-mm-diameter plunger. Two measurements were taken on each apple on opposite sides at the equatorial region, after removing a thin disc of skin. Total soluble solids, as an indicator of soluble sugar content, were determined with a refractometer (Atago, ATC-1; McCormick Fruit Tech, Yakima, WA, USA). Skin color was determined using a colorimeter (PCE-SM2) via two measurements on opposite sides of the fruit, in the equatorial region. Color variation was calculated based on the CIE L*a*b* color space parameters [90], where L* is the lightness coordinate, a* represents the red (+a*)–green (−a*) axis, and b* represents the yellow (+b*)–blue (−b*) axis.

The following equation was used to calculate the total color variation:

ΔE*ab = [(L*t_0_ − L*t_n_)^2^ + (a*t_0_ − a*t_n_)^2^ + (b*t_0_ − b*t_n_)^2^] ^1/2^

where t_0_ is the day of the initial measurement and t_n_ is each of the subsequent measurement days.

#### 4.2.2. Ethylene Production

Ethylene production was determined by GC-FID, as described by Verde et al. [59]. Apple samples were individually placed in hermetically sealed containers fitted with septa and then incubated for 1 h in the dark at 25 °C. After incubation, 1 mL of the headspace was extracted and injected into a gas chromatograph (Hewlett-Packard 6890 Plus, Agilent Technologies, Santa Clara, CA, USA). Ten individual samples were used to measure the ethylene released at each time point of the study. Ethylene production was expressed as µL kg^−1^ h^−1^.

#### 4.2.3. Phenolic Content

Polyphenols were extracted from apple skin and flesh samples using a method adapted from Jakobek et al. [91]. Briefly, 0.5 g of each sample was homogenized using a mortar in 5 mL of solvent: 80% methanol for the flesh and acidified 80% methanol (1% HCl) for the skin. The homogenates were vortexed and subjected to ultrasonic extraction for 15 min at room temperature, then centrifuged at 3000× *g* for 5 min at 4 °C. The supernatant was collected, and the pellet was re-extracted with an additional 2 mL of the respective solvent. Both supernatants were combined and stored at −80 °C until analysis.

Total phenolic content (TPC) was determined according to the Folin–Ciocalteu method, with gallic acid (Sigma-Aldrich, St. Louis, MO, USA) as the standard [92], with minor modifications. Briefly, 100 µL of skin extract (diluted 1:5, *v*/*v*, in distilled water) or undiluted flesh extract was mixed with 0.5 mL of 10% Folin–Ciocalteu reagent and allowed to react for 5 min in the dark at room temperature. Subsequently, 1.4 mL of 7.5% Na_2_CO_3_ was added and the mixture was incubated for 1 h in the dark at room temperature. Absorbance was measured at 765 nm. Results were expressed as µg gallic acid equivalents per gram of fresh weight (µg GAE/g FW).

Total flavonoid content (TFC) was determined colorimetrically [93]. Skin extracts were diluted 1:5 (*v*/*v*) in distilled water; flesh extracts were used undiluted. After vortexing, 60 µL of 5% NaNO_2_ was added and the mixture was incubated for 5 min in the dark at room temperature. Then 120 µL of 10% AlCl_3_ was added and the mixture was incubated for a further 6 min in the dark, followed by the addition of 400 µL of 1 N NaOH and 220 µL of distilled water. After vortexing, the mixture was kept for 20 min in the dark at room temperature, and absorbance was measured at 510 nm. Results were expressed as µg catechin equivalents per gram of fresh weight (µg CE/g FW), using (+)-catechin (Merck, Darmstadt, Germany) as the standard.

#### 4.2.4. Determination of the Polyphenolic Profile

The polyphenol profile in skin and flesh extracts was determined using HPLC-DAD, according to the method of Kahle et al. [94] with modifications. The equipment consisted of a Hewlett-Packard 1100 series HPLC equipped with a quaternary pump (HP G1311A), an in-line degasser (HP G1322A), a 20 µL injection valve (HP C13228A), and a diode array detector (G4212A 1290 DAD). Detection wavelengths were set at 254, 280, and 320 nm, with spectral acquisition from 200 to 600 nm. Chromatographic separation was performed on a reverse-phase C18 column (Supelco Supelcosil LC-18-DB (Merk, Germany), 150 mm × 4.6 mm, 5 µm particle size), maintained at 30 °C. The mobile phase consisted of (A) 0.1% formic acid in Milli-Q water and (B) 0.1% formic acid in acetonitrile (100%), filtered through a 0.45-µm membrane and degassed under vacuum. The flow rate was 1 mL/min. The gradient program was as follows: min 0, A (90%)–B (10%); min 20, A (70%)–B (30%); min 22, A (70%)–B (30%); min 25, A (90%)–B (10%).

Chromatographic data were recorded, and peak areas were integrated using ChemStation software B.04.03. Polyphenols were identified and quantified by comparing their retention times and UV–vis spectra (200–600 nm) with those of authentic external standards (0.001–0.1 mg/mL), injected in triplicate at the beginning of each analytical session and repeated every four samples. Procyanidin B2, (−)-epicatechin, chlorogenic acid, *o*-coumaric acid, caffeic acid, *p*-coumaric acid, phloridzin, rutin and quercetin were obtained from Sigma-Aldrich (St. Louis, MO, USA), and (+)-catechin and avicularin from Merck (Darmstadt, Germany); each compound was quantified against its own authentic standard. In addition, the standards hyperoside and isoquercetin (Merck, Darmstadt, Germany) and *trans*-cinnamic acid and phloretin (Sigma-Aldrich, St. Louis, MO, USA) were assayed but not detected in the samples, and were therefore not included in the analysis. Individual polyphenol contents were expressed as µg per gram of skin or flesh fresh weight (µg/g FW).

### 4.3. Statistical Analysis

All statistical analyses were performed using R software R-4.6.1 and GraphPad Prism v8.0.1. Within each group, data normality was assessed using the Shapiro–Wilk test; non-normally distributed data were log-transformed prior to further analysis. Homogeneity of variances was evaluated using Levene’s test on the transformed data. The ripening evolution of control fruit was analyzed separately by one-way ANOVA with sampling time (0, 7, 14, and 35 days) as the main factor. For treatment effects, data from days 7, 14, and 35 were analyzed using a three-way factorial ANOVA considering melatonin treatment (MEL, four levels: 0, 10, 50, and 500 µM), ACC treatment (ACC, two levels: absent/present), and sampling time (three levels: 7, 14, and 35 days) as fixed factors, including all possible interactions among factors. In addition, within each sampling time, differences among treatment groups were assessed by one-way ANOVA followed by Tukey’s multiple comparison test. Statistical significance was established at *p* < 0.05.

## 5. Conclusions

Exogenous melatonin application is confirmed as a physiological modulator of postharvest ripening in ‘Reineta’ apple (*Malus domestica*) with a dual behavior: it promotes the climacteric transition—advancing ethylene production and accelerating sugar accumulation and the development of the cultivar’s color—and, simultaneously, preserves fruit firmness and sustains the content of phenolics, flavonoids and individual polyphenols, especially in the skin and most markedly at the 50 and 500 µM doses. The uncoupling between ethylene stimulation and firmness retention, together with the fact that ACC reproduced the ethylene response without modifying texture or the phenolic profile, indicates that the action of melatonin is not channeled solely through the ethylene pathway but incorporates an autonomous protective component, plausibly linked to its antioxidant capacity and to the stabilization of the cell wall and membranes; this component persists even when melatonin is combined with ACC and the climacteric signal is amplified. The selective reinforcement of chlorogenic acid, procyanidin B2 and quercetin derivatives—antioxidants with affinity for the cell wall—may contribute to the greater retained firmness, although a direct causal relationship cannot be established from the present data. Overall, melatonin coordinately improves organoleptic (sweetness and color), mechanical (firmness) and nutritional (polyphenolic profile) attributes of the fruit during ripening at ambient temperature, identifying this indoleamine as a promising agent for postharvest ripening control in apples.

## Figures and Tables

**Figure 1:**
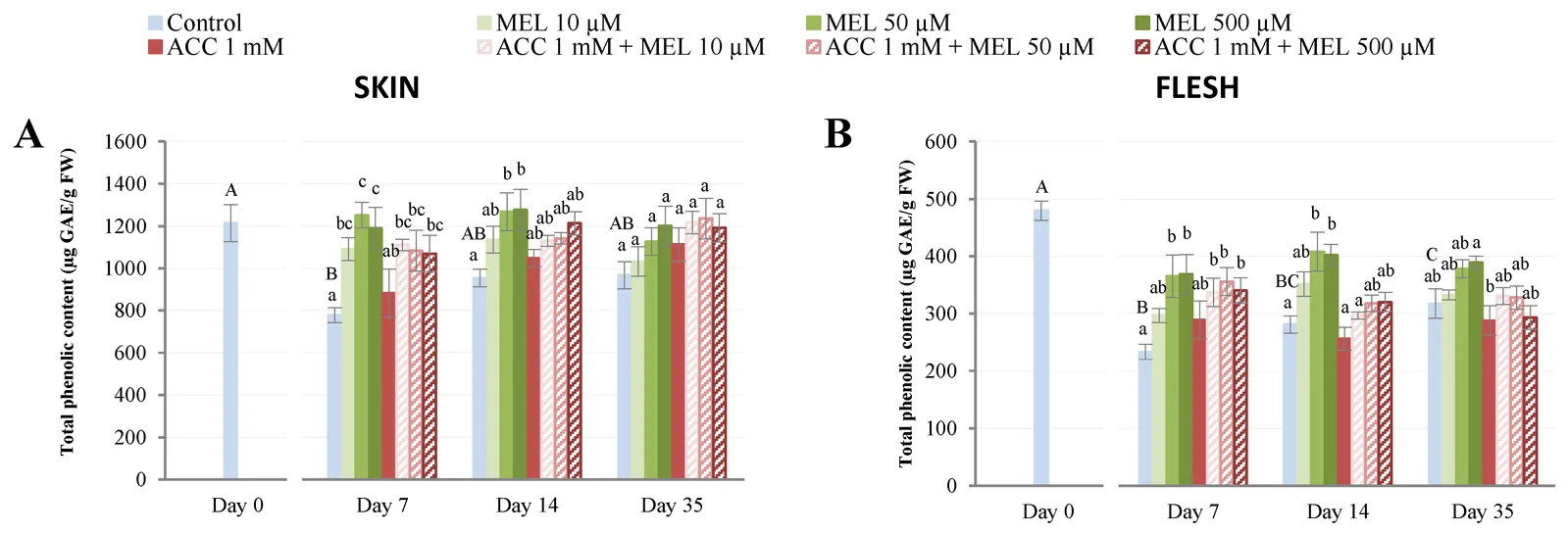
Total phenolic content (µg GAE/g FW) during the postharvest period in ‘Reineta’ apples treated with melatonin (MEL), ACC, and their combination (MEL + ACC), in (**A**) skin and (**B**) flesh. Statistics: different capital letters indicate significant differences over time for the control group, *p* < 0.05; different lowercase letters indicate significant differences between treatment groups within the same time, *p* < 0.05.

**Figure 2:**
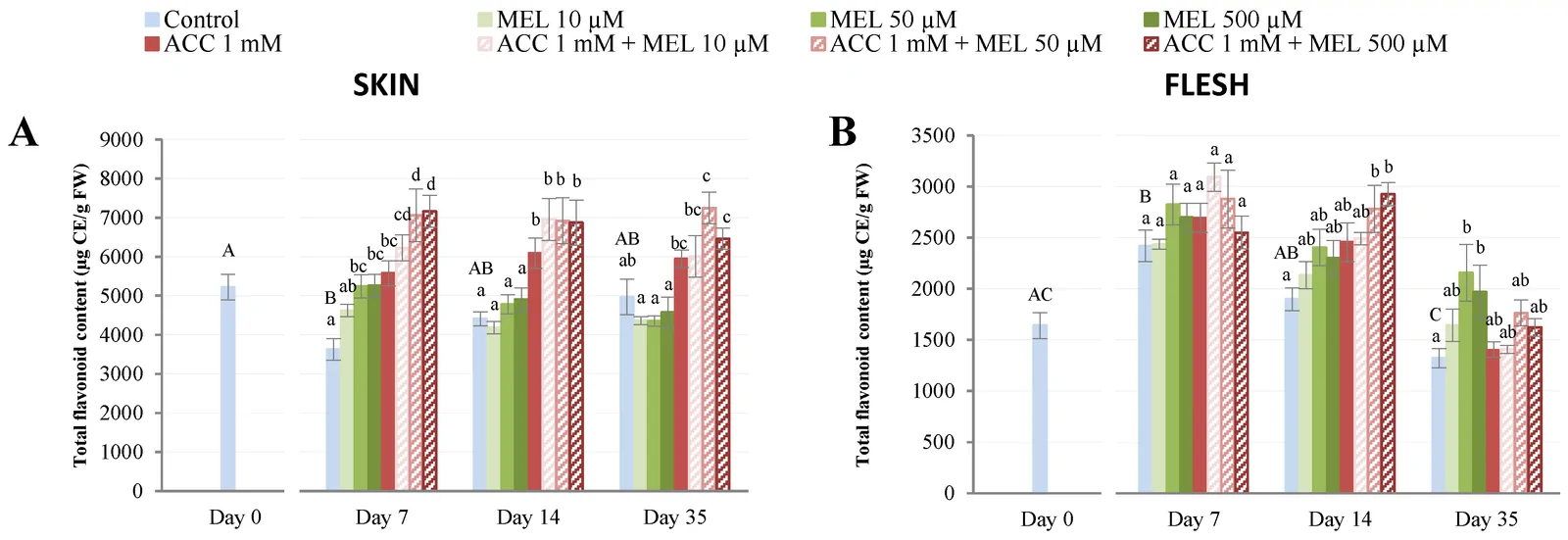
Total flavonoid content (µg CE/g FW) during the postharvest period in ‘Reineta’ apples treated with melatonin (MEL), ACC, and their combination (MEL + ACC), in (**A**) skin and (**B**) flesh. Statistics: different capital letters indicate significant differences over time for the control group, *p* < 0.05; different lowercase letters indicate significant differences between treatment groups within the same time, *p* < 0.05.

**Figure 4:**
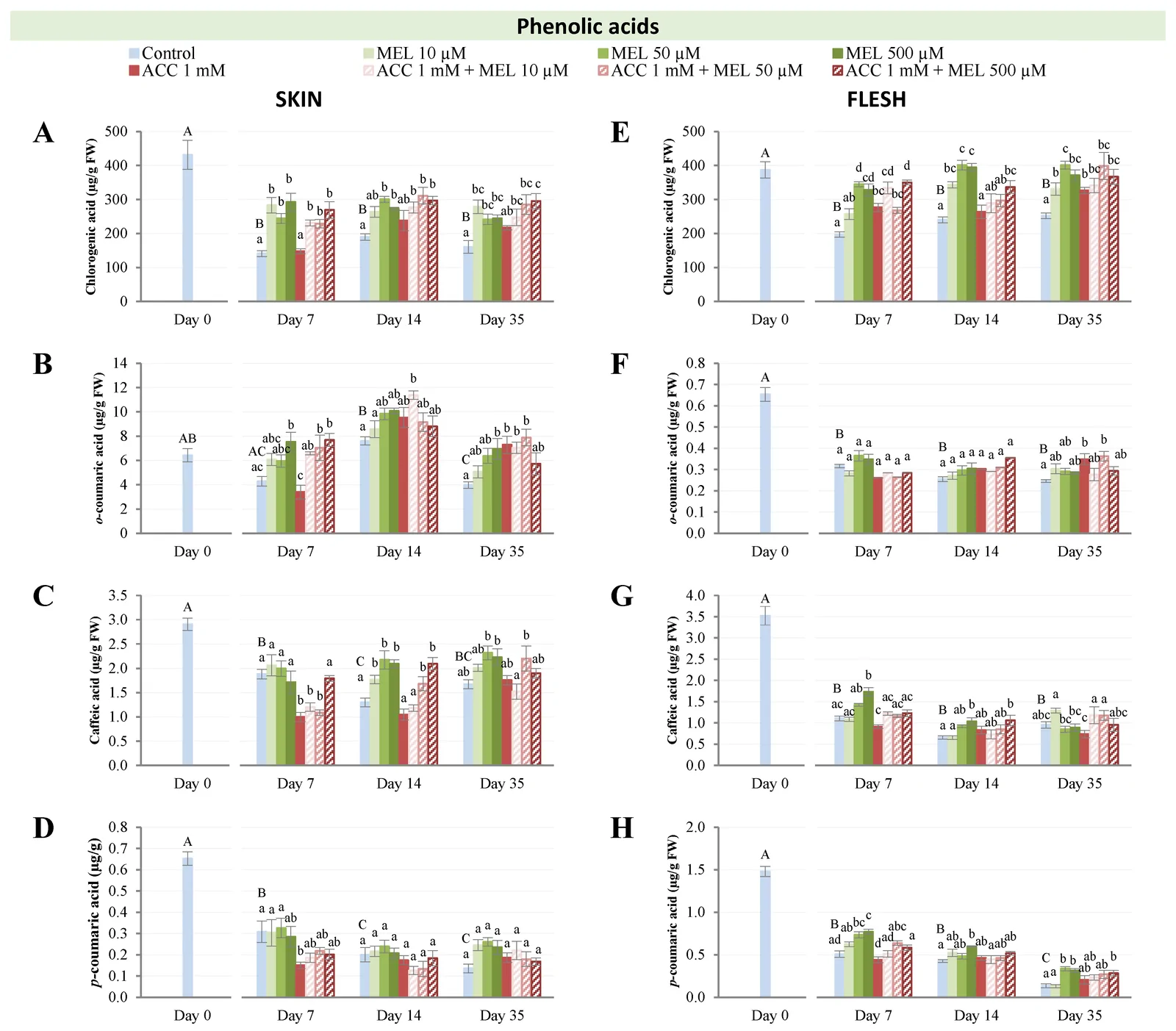
Polyphenolic profile of the phenolic acids group (µg/g FW) in skin (**A**–**D**) and flesh (**E**–**H**) samples of ‘Reineta’ apples treated with melatonin (MEL), ACC, and their combination (MEL + ACC) during the postharvest period. Identified compounds: (**A**,**E**) chlorogenic, (**B**,**F**) *o*-coumaric, (**C**,**G**) caffeic, and (**D**,**H**) *p*-coumaric acids. Statistics: different capital letters indicate significant differences over time for the control group, *p* < 0.05; different lowercase letters indicate significant differences between treatment groups within the same time, *p* < 0.05.

**Figure 5:**
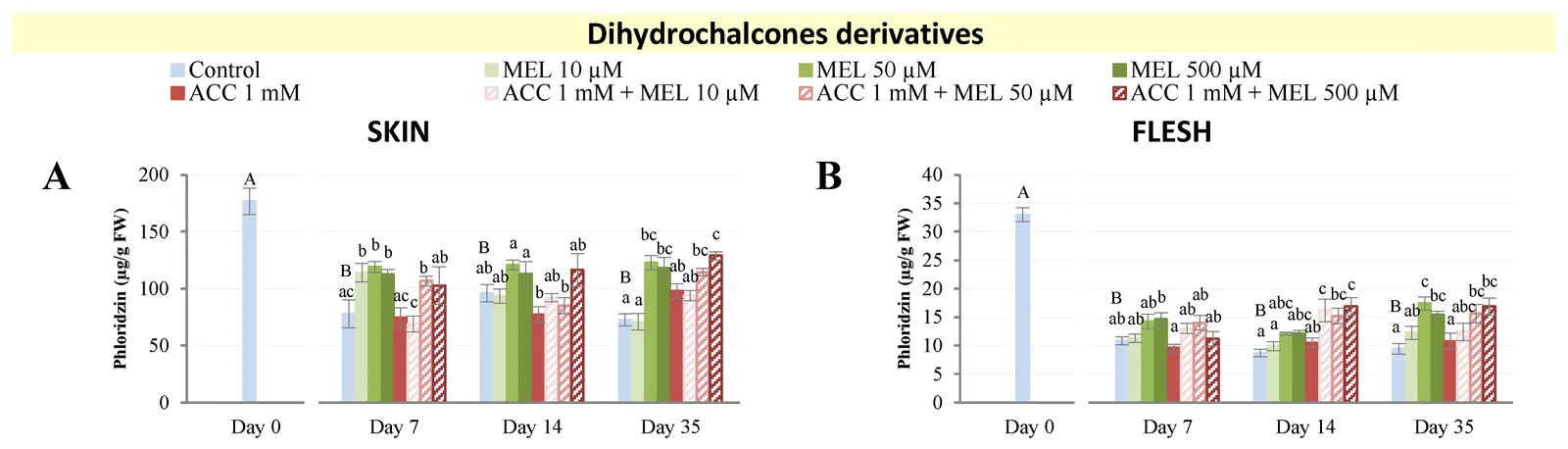
Polyphenolic profile of the dihydrochalcone derivatives group (µg/g FW) in skin (**A**) and flesh (**B**) samples of ‘Reineta’ apples treated with melatonin (MEL), ACC, and their combination (MEL + ACC) during the postharvest period. Identified compound: phloridzin. Statistics: different capital letters indicate significant differences over time for the control group, *p* < 0.05; different lowercase letters indicate significant differences between treatment groups within the same time, *p* < 0.05.

**Figure 6:**
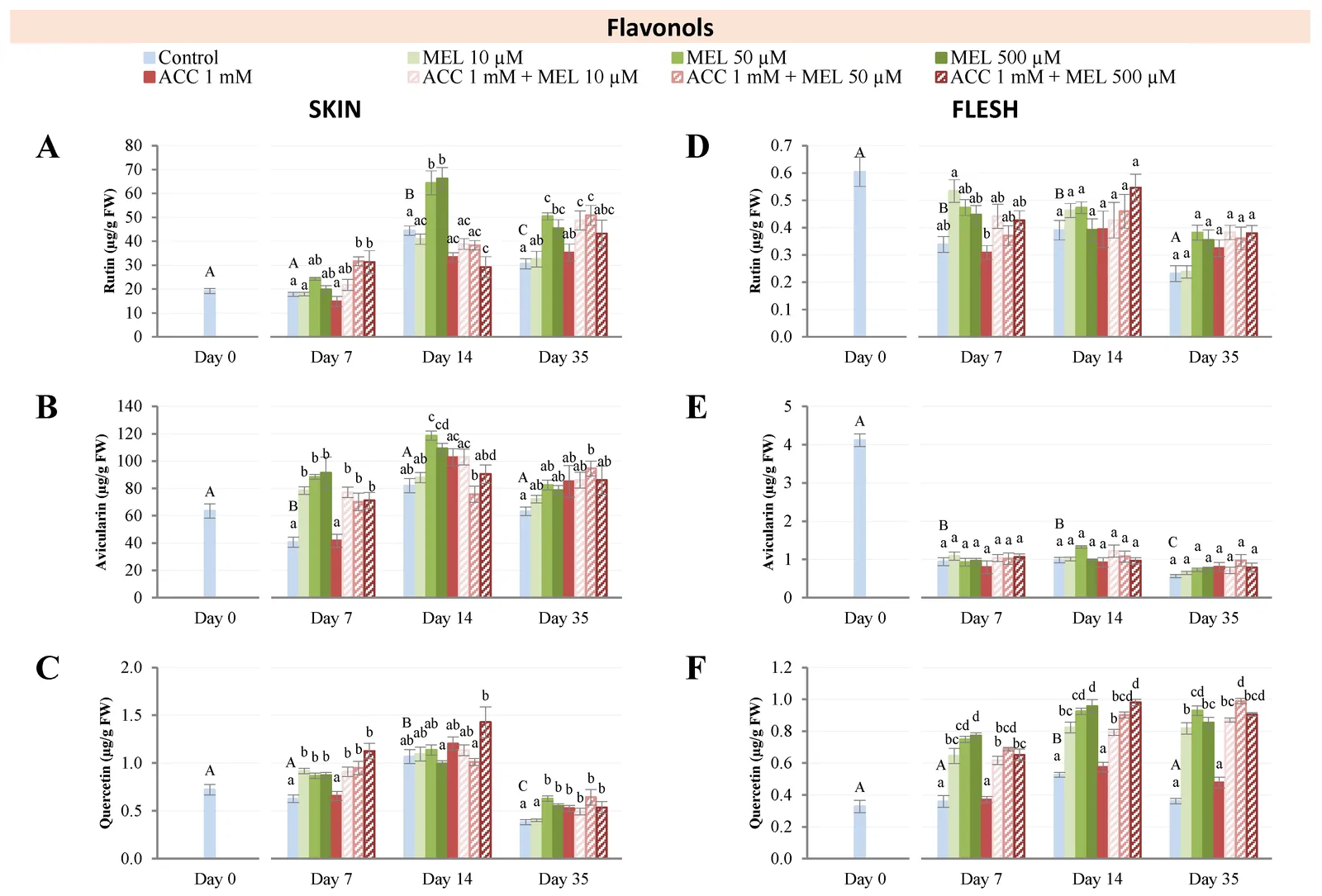
Polyphenolic profile of the flavonol group (µg/g FW) in skin (**A**–**C**) and flesh (**D**–**F**) samples of ‘Reineta’ apples treated with melatonin (MEL), ACC, and their combination (MEL + ACC) during the postharvest period. Identified compounds: (**A**,**D**) rutin, (**B**,**E**) avicularin, and (**C**,**F**) quercetin. Statistics: different capital letters indicate significant differences over time for the control group, *p* < 0.05; different lowercase letters indicate significant differences between treatment groups within the same time, *p* < 0.05.

**Table 1:** Evolution of sugar content, firmness, color variation, and ethylene production during the postharvest period in ‘Reineta’ apples treated with melatonin (MEL), ACC and their combination (MEL + ACC).

	Day 0	Day 7		Day 14		Day 35	
***Sugar content (ºBrix)***							
**Control**	10.35 ± 0.15 **^A^**	11.75 ± 0.21	**^B,a^**	12.68 ± 0.36	**^C,ab^**	13.40 ± 0.32	**^D,a^**
**MEL 10 µM**	-	12.05 ± 0.26	**^A,a^**	13.25 ± 0.10	**^B,b^**	13.95 ± 0.10	**^B,ab^**
**MEL 50 µM**	-	12.08 ± 0.19	**^A,a^**	13.20 ± 0.23	**^B,ab^**	14.53 ± 0.05	**^C,b^**
**MEL 500 µM**	-	11.85 ± 0.10	**^A,a^**	12.90 ± 0.29	**^B,ab^**	13.60 ± 0.29	**^B,a^**
**ACC 1 mM**	-	10.45 ± 0.10	**^A,b^**	12.30 ± 0.14	**^B,a^**	13.43 ± 0.17	**^C,a^**
**ACC 1 mM + MEL 10 µM**	-	11.78 ± 0.29	**^A,a^**	13.65 ± 0.25	**^B,b^**	13.85 ± 0.25	**^B,ab^**
**ACC 1 mM + MEL 50 µM**	-	11.70 ± 0.29	**^A,a^**	13.38 ± 0.15	**^B,b^**	14.60 ± 0.14	**^C,b^**
**ACC 1 mM + MEL 500 µM**	-	11.73 ± 0.05	**^A,a^**	12.93 ± 0.15	**^B,ab^**	14.18 ± 0.12	**^C,ab^**
***Firmness* (kg/cm^2^)**							
**Control**	19.95 ± 0.64 **^A^**	17.68 ± 0.64	**^B,ab^**	15.56 ± 0.31	**^C,a^**	10.86 ± 0.34	**^d,a^**
**MEL 10 µM**	-	18.96 ± 0.40	**^A,b^**	15.81 ± 0.22	**^B,a^**	12.62 ± 0.19	**^C,b^**
**MEL 50 µM**	-	19.40 ± 0.22	**^A,b^**	16.71 ± 0.23	**^B,a^**	13.35 ± 0.31	**^C,b^**
**MEL 500 µM**	-	18.04 ± 0.24	**^A,ab^**	16.00 ± 0.58	**^B,a^**	13.79 ± 0.37	**^C,b^**
**ACC 1 mM**	-	17.19 ± 0.50	**^A,a^**	15.46 ± 0.29	**^B,a^**	10.13 ± 0.14	**^C,a^**
**ACC 1 mM + MEL 10 µM**	-	18.79 ± 0.29	**^A,b^**	15.92 ± 0.30	**^B,a^**	12.83 ± 0.28	**^C,b^**
**ACC 1 mM + MEL 50 µM**	-	18.04 ± 0.31	**^A,ab^**	15.42 ± 0.42	**^B,a^**	13.80 ± 0.34	**^C,b^**
**ACC 1 mM + MEL 500 µM**	-	17.92 ± 0.28	**^A,ab^**	15.29 ± 0.35	**^B,a^**	14.07 ± 0.63	**^B,b^**
*Color variation* (Δ*Eab* *)							
**Control**	n.a.	6.20 ± 0.84	**^A,a^**	6.85 ± 0.50	**^A,a^**	15.54 ± 0.36	**^B,a^**
**MEL 10 µM**	-	10.62 ± 0.82	**^A,b^**	15.21 ± 0.68	**^B,b^**	26.54 ± 0.56	**^C,b^**
**MEL 50 µM**	-	14.26 ± 0.67	**^A,c^**	15.41 ± 0.70	**^A,b^**	24.70 ± 0.86	**^B,b^**
**MEL 500 µM**	-	10.90 ± 0.35	**^A,b^**	12.02 ± 0.39	**^A,c^**	21.79 ± 0.73	**^B,d^**
**ACC 1 mM**	-	11.29 ± 0.80	**^A,b^**	12.99 ± 0.57	**^A,bc^**	18.88 ± 0.53	**^B,e^**
**ACC 1 mM + MEL 10 µM**	-	12.69 ± 0.41	**^A,bc^**	13.87 ± 0.40	**^A,bc^**	23.86 ± 0.67	**^B,bd^**
**ACC 1 mM + MEL 50 µM**	-	11.32 ± 0.74	**^A,b^**	14.38 ± 0.46	**^B,bc^**	23.68 ± 0.87	**^C,bd^**
**ACC 1 mM + MEL 500 µM**	-	12.71 ± 0.53	**^A,bc^**	13.25 ± 0.50	**^A,bc^**	24.49 ± 0.46	**^B,bd^**
***Ethylene production* (µL·kg^−1^·h^−1^)**							
**Control**	n.d.	n.d.		8.95 ± 0.01	**^A,a^**	85.94 ± 1.55	**^B,a^**
**MEL 10 µM**	-	5.10 ± 0.08	**^A,a^**	57.45 ± 4.57	**^B,bc^**	105.39 ± 3.94	**^C,b^**
**MEL 50 µM**	-	4.80 ± 0.13	**^A,a^**	56.81 ± 3.30	**^B,bc^**	106.19 ± 4.95	**^C,b^**
**MEL 500 µM**	-	6.06 ± 0.12	**^A,ab^**	43.98 ± 2.43	**^B,b^**	121.99 ± 3.46	**^C,bc^**
**ACC 1 mM**	-	12.50 ± 0.79	**^A,bc^**	60.16 ± 4.44	**^B,c^**	106.98 ± 7.51	**^C,b^**
**ACC 1 mM + MEL 10 µM**	-	13.31 ± 0.26	**^A,c^**	93.65 ± 3.14	**^B,d^**	132.76 ± 9.92	**^C,cd^**
**ACC 1 mM + MEL 50 µM**	-	4.45 ± 0.27	**^A,a^**	90.36 ± 4.73	**^B,d^**	141.07 ± 3.36	**^C,d^**
**ACC 1 mM + MEL 500 µM**	-	5.45 ± 0.55	**^A,a^**	115.57 ± 6.42	**^B,e^**	125.11 ± 7.30	**^B,bcd^**

* n.a.: not applicable; n.d.: not detectable; Statistics: different capital letters indicate significant differences over time within the same treatment group, *p* < 0.05; different lowercase letters indicate significant differences between treatment groups within the same time, *p* < 0.05.

**Table 2:** Statistical significance (*p*-values) of main-factor effects and interactions from the three-way ANOVA for total phenolic and flavonoid contents in the skin and flesh of ‘Reineta’ apples.

	SKIN		FLESH	
	Phenols	Flavonoids	Phenols	Flavonoids
**Time**	0.028	0.708	0.665	<0.001
**ACC**	0.632	<0.001	<0.001	0.030
**MEL**	<0.001	<0.001	<0.001	<0.001
**Time × ACC**	0.076	0.646	0.002	<0.001
**Time × MEL**	0.484	0.119	0.536	0.432
**ACC × MEL**	0.072	0.389	0.021	0.351
**Time × ACC × MEL**	0.917	0.407	0.952	0.412

**Table 3:** Contribution of the main polyphenolic categories to the total polyphenolic content of ‘Reineta’ apple skin (µg/g FW) during the postharvest period studied.

Polyphenolic Group	Day 0			Day 7			Day 14			Day 35		
**Control**												
Σ Flavanols	1782.62	±	77.88**^A^**	831.68	±	47.70**^B^**	1356.04	±	77.96**^C^**	1351.84	±	26.47**^C^**
Σ Phenolic acids	441.26	±	43.08**^A^**	147.43	±	8.39**^B^**	198.68	±	9.76**^B^**	166.39	±	18.48**^B^**
Σ Dihydrochalcones	176.61	±	11.66**^A^**	77.83	±	12.08**^B^**	95.96	±	7.58**^B^**	72.45	±	5.35**^B^**
Σ Flavonols	83.23	±	5.02**^A^**	59.04	±	4.32**^B^**	127.49	±	6.09**^C^**	94.13	±	3.59**^D^**
**Total**	**2483.72**	**±**	**125.76 ^A^**	**1115.98**	**±**	**52.76 ^B,a^**	**1778.17**	**±**	**84.67 ^C,a^**	**1684.81**	**±**	**35.43 ^C,a^**
**MEL 10 µM**												
Σ Flavanols		**-**		830.63	±	52.30**^A^**	1400.64	±	95.85**^B^**	1341.45	±	91.83**^B^**
Σ Phenolic acids		**-**		291.57	±	23.24**^A^**	274.30	±	15.77**^A^**	285.80	±	19.93**^A^**
Σ Dihydrochalcones		**-**		113.90	±	8.16**^A^**	93.41	±	6.15**^AB^**	70.75	±	7.37**^B^**
Σ Flavonols		**-**		97.23	±	3.00**^A^**	129.90	±	5.02**^B^**	105.11	±	4.35**^C^**
**Total**		**-**		**1333.33**	**±**	**38.95 ^A,a^**	**1898.25**	**±**	**95.43 ^B,ab^**	**1803.11**	**±**	**84.49 ^B,ab^**
**MEL 50 µM**												
Σ Flavanols		**-**		1172.68	±	32.15**^A^**	1810.97	±	72.62**^B^**	1502.77	±	50.47**^C^**
Σ Phenolic acids		**-**		252.75	±	14.92**^A^**	312.87	±	7.95**^B^**	250.50	±	15.71**^A^**
Σ Dihydrochalcones		**-**		119.13	±	4.92**^A^**	120.80	±	4.26**^A^**	122.74	±	6.24**^A^**
Σ Flavonols		**-**		113.71	±	2.33**^A^**	184.19	±	4.98**^B^**	133.51	±	3.41**^C^**
**Total**		**-**		**1658.27**	**±**	**36.48 ^A,b^**	**2428.83**	**±**	**73.23 ^B,c^**	**2009.51**	**±**	**53.61 ^C,bc^**
**MEL 500 µM**												
Σ Flavanols		**-**		1334.73	±	69.40**^A^**	1774.04	±	78.70**^B^**	1605.21	±	47.18**^B^**
Σ Phenolic acids		**-**		243.72	±	61.78**^A^**	285.26	±	4.51**^A^**	253.77	±	9.93**^A^**
Σ Dihydrochalcones		**-**		112.43	±	4.25**^A^**	112.90	±	10.98**^A^**	118.18	±	9.20**^A^**
Σ Flavonols		**-**		112.03	±	11.96**^A^**	176.48	±	6.73**^B^**	124.63	±	6.18**^C^**
**Total**		**-**		**1802.91**	**±**	**103.62 ^A,bc^**	**2348.67**	**±**	**86.88 ^B,c^**	**2101.80**	**±**	**55.10 ^B,bc^**
**ACC 1 mM**												
Σ Flavanols		**-**		938.98	±	101.25**^A^**	1492.50	±	112.34**^B^**	1711.36	±	100.68**^B^**
Σ Phenolic acids		**-**		151.99	±	7.29**^A^**	248.72	±	29.11**^B^**	226.21	±	6.17**^B^**
Σ Dihydrochalcones		**-**		74.46	±	8.65**^A^**	77.31	±	7.01**^A^**	98.29	±	6.45**^A^**
Σ Flavonols		**-**		57.18	±	5.33**^A^**	137.28	±	7.08**^B^**	120.94	±	10.15**^B^**
**Total**				**1222.61**	**±**	**100.43 ^A,a^**	**1955.81**	**±**	**124.28 ^B,ab^**	**2156.79**	**±**	**94.88 ^B,bc^**
**ACC 1 mM + MEL 10 µM**												
Σ Flavanols		**-**		1267.35	±	14.30**^A^**	1652.85	±	111.51**^B^**	1952.85	±	77.91**^C^**
Σ Phenolic acids		**-**		238.99	±	8.47**^A^**	289.32	±	15.99**^A^**	256.23	±	24.92**^A^**
Σ Dihydrochalcones		**-**		68.84	±	7.08**^A^**	92.02	±	3.52**^B^**	94.03	±	4.53**^B^**
Σ Flavonols		**-**		99.81	±	3.95**^A^**	142.96	±	6.63**^B^**	135.32	±	5.84**^B^**
**Total**		**-**		**1674.98**	**±**	**8.97 ^A,b^**	**2177.16**	**±**	**102.65 ^B,bc^**	**2438.42**	**±**	**97.27 ^C,cd^**
**ACC 1 mM + MEL 50 µM**												
Σ Flavanols		**-**		1554.84	±	44.63**^A^**	1689.03	±	79.71**^AB^**	1866.72	±	63.85**^B^**
Σ Phenolic acids		**-**		237.28	±	13.20**^A^**	322.05	±	24.37**^B^**	295.42	±	28.66**^B^**
Σ Dihydrochalcones		**-**		106.71	±	4.19**^AB^**	85.13	±	7.22**^A^**	114.52	±	3.08**^B^**
Σ Flavonols		**-**		102.79	±	8.11**^A^**	115.09	±	6.34**^A^**	146.08	±	9.19**^B^**
**Total**		**-**		**2001.62**	**±**	**55.17 ^A,c^**	**2211.29**	**±**	**90.55 ^AB,bc^**	**2422.73**	**±**	**94.41 ^B,cd^**
**ACC 1 mM + MEL 500 µM**												
Σ Flavanols		**-**		1515.45	±	91.79**^A^**	1660.43	±	36.45**^A^**	2157.16	±	73.98**^B^**
Σ Phenolic acids		**-**		278.85	±	24.56**^A^**	307.91	±	11.94**^A^**	302.93	±	22.29**^A^**
Σ Dihydrochalcones		**-**		102.82	±	16.54**^A^**	116.48	±	14.31**^A^**	128.95	±	3.48**^A^**
Σ Flavonols		**-**		103.54	±	6.04**^A^**	121.14	±	8.56**^AB^**	129.80	±	15.53**^B^**
**Total**		**-**		**2000.65**	**±**	**72.11 ^A,c^**	**2205.96**	**±**	**27.71 ^A,bc^**	**2718.84**	**±**	**74.27 ^B,d^**

This index is calculated as the sum of the various individual components within each category and time interval (mean ± SE). Statistics: different capital letters indicate significant differences over time within the same treatment group, *p* < 0.05; different lowercase letters indicate significant differences between treatment groups within the same time, *p* < 0.05.

**Table 4:** Contribution of the main polyphenolic categories to the total polyphenolic content of ‘Reineta’ apple flesh (µg/g FW) during the postharvest period studied.

Polyphenolic Group	Day 0			Day 7			Day 14			Day 35		
**Control**												
Σ Flavanols	413.70	±	9.32**^A^**	130.91	±	10.89**^B^**	118.91	±	6.15**^B^**	149.18	±	8.60**^B^**
Σ Phenolic acids	392.72	±	24.34**^A^**	198.98	±	7.63**^B^**	241.78	±	8.79**^B^**	253.48	±	8.30**^B^**
Σ Dihydrochalcones	32.98	±	1.22**^A^**	10.81	±	0.70**^B^**	8.64	±	0.65**^B^**	9.36	±	0.93**^B^**
Σ Flavonols	4.97	±	0.19**^A^**	1.64	±	0.09**^BC^**	1.90	±	0.10**^B^**	1.16	±	0.04**^C^**
**Total**	**844.37**	**±**	**31.33 ^A^**	**342.34**	**±**	**9.84 ^B,a^**	**371.23**	**±**	**12.32 ^B,a^**	**413.19**	**±**	**15.74 ^B,a^**
**MEL 10 µM**												
Σ Flavanols		**-**		134.00	±	5.65**^A^**	200.02	±	10.94**^B^**	167.12	±	4.55**^AB^**
Σ Phenolic acids		**-**		259.05	±	15.71**^A^**	344.31	±	9.14**^B^**	332.64	±	18.22**^B^**
Σ Dihydrochalcones		**-**		11.27	±	0.75**^A^**	9.90	±	0.81**^A^**	12.24	±	1.16**^A^**
Σ Flavonols		**-**		2.27	±	0.11**^A^**	2.30	±	0.03**^A^**	1.71	±	0.09**^B^**
**Total**		**-**		**406.59**	**±**	**19.64 ^A,ab^**	**556.52**	**±**	**19.75 ^B,dc^**	**513.70**	**±**	**16.01 ^B,b^**
**MEL 50 µM**												
Σ Flavanols		**-**		198.04	±	7.11**^A^**	227.66	±	13.73**^A^**	211.13	±	8.54**^A^**
Σ Phenolic acids		**-**		347.19	±	7.62**^A^**	402.80	±	13.80**^A^**	402.45	±	11.53**^A^**
Σ Dihydrochalcones		**-**		14.22	±	1.26**^AB^**	12.08	±	0.27**^A^**	17.42	±	1.15**^B^**
Σ Flavonols		**-**		2.15	±	0.08**^A^**	2.73	±	0.05**^B^**	2.04	±	0.07**^A^**
**Total**		**-**		**561.60**	**±**	**13.50 ^A,d^**	**645.27**	**±**	**25.38 ^B,d^**	**633.04**	**±**	**9.15 ^AB,cd^**
**MEL 500 µM**												
Σ Flavanols		**-**		189.54	±	7.26**^A^**	218.53	±	11.51**^A^**	202.63	±	8.01**^A^**
Σ Phenolic acids		**-**		331.10	±	17.56**^A^**	396.70	±	12.41**^B^**	372.60	±	16.19**^AB^**
Σ Dihydrochalcones		**-**		14.63	±	1.14**^A^**	12.11	±	0.42**^A^**	15.41	±	0.58**^A^**
Σ Flavonols		**-**		2.18	±	0.09**^A^**	2.33	±	0.06**^A^**	1.96	±	0.07**^A^**
**Total**		**-**		**537.46**	**±**	**22.74 ^A,cd^**	**629.67**	**±**	**23.26 ^B,de^**	**592.60**	**±**	**20.01 ^AB,bcd^**
**ACC 1 mM**												
Σ Flavanols		**-**		163.61	±	11.31**^A^**	117.07	±	11.79**^B^**	182.46	±	11.05**^A^**
Σ Phenolic acids		**-**		278.26	±	12.05**^A^**	265.11	±	19.83**^A^**	327.73	±	9.28**^B^**
Σ Dihydrochalcones		**-**		9.55	±	0.67**^A^**	10.44	±	0.89**^A^**	10.94	±	1.39**^A^**
Σ Flavonols		**-**		1.48	±	0.16**^A^**	1.89	±	0.15**^B^**	1.61	±	0.13**^AB^**
**Total**				**452.90**	**±**	**16.19 ^AB,bc^**	**394.51**	**±**	**25.43 ^A,ab^**	**522.74**	**±**	**6.91 ^B,b^**
**ACC 1 mM + MEL 10 µM**												
Σ Flavanols		**-**		213.33	±	10.33**^A^**	160.68	±	16.14**^B^**	188.19	±	8.57**^AB^**
Σ Phenolic acids		**-**		336.06	±	17.15**^A^**	290.61	±	27.66**^A^**	343.00	±	21.00**^A^**
Σ Dihydrochalcones		**-**		13.03	±	0.87**^A^**	16.15	±	1.98**^A^**	12.51	±	1.54**^A^**
Σ Flavonols		**-**		2.09	±	0.11**^AB^**	2.44	±	0.19**^A^**	1.97	±	0.10**^B^**
**Total**		**-**		**564.50**	**±**	**18.73 ^A,d^**	**469.89**	**±**	**36.71 ^B,bc^**	**545.67**	**±**	**25.60 ^A,bc^**
**ACC 1 mM + MEL 50 µM**												
Σ Flavanols		**-**		214.41	±	11.27**^A^**	170.30	±	8.10**^B^**	237.66	±	17.07**^A^**
Σ Phenolic acids		**-**		269.93	±	8.60**^A^**	298.21	±	18.09**^A^**	400.65	±	39.50**^B^**
Σ Dihydrochalcones		**-**		14.02	±	1.30**^A^**	15.17	±	1.38**^A^**	15.65	±	1.62**^A^**
Σ Flavonols		**-**		2.08	±	0.17**^A^**	2.44	±	0.17**^A^**	2.32	±	0.17**^A^**
**Total**		**-**		**500.44**	**±**	**20.78 ^A,cd^**	**486.11**	**±**	**25.62 ^A,bc^**	**656.27**	**±**	**45.65 ^B,d^**
**ACC 1 mM + MEL 500 µM**												
Σ Flavanols		**-**		216.34	±	2.97**^A^**	185.93	±	10.79**^A^**	214.21	±	14.88**^A^**
Σ Phenolic acids		**-**		352.30	±	7.05**^A^**	338.68	±	18.62**^A^**	368.33	±	21.85**^A^**
Σ Dihydrochalcones		**-**		11.17	±	1.28**^A^**	17.01	±	1.54**^B^**	16.86	±	1.53**^B^**
Σ Flavonols		**-**		2.14	±	0.09**^A^**	2.49	±	0.07**^B^**	2.07	±	0.10**^A^**
**Total**		**-**		**581.95**	**±**	**9.99 ^A,d^**	**544.12**	**±**	**25.90 ^A,ce^**	**601.47**	**±**	**34.92 ^A,bcd^**

This index is calculated as the sum of the various individual components within each category and time interval (mean ± SE). Statistics: different capital letters indicate significant differences over time within the same treatment group, *p* < 0.05; different lowercase letters indicate significant differences between treatment groups within the same time, *p* < 0.05.

## Data Availability

The data presented in this study are openly available in Zenodo at https://doi.org/10.5281/zenodo.22832082, reference number 10.5281/zenodo.22832082.
